# Reconfiguring biofortification strategies to transform food systems and address micronutrient deficiency of the 21st century

**DOI:** 10.1111/jipb.70305

**Published:** 2026-06-08

**Authors:** Rhowell Jr. N. Tiozon, Alisdair R. Fernie, Nese Sreenivasulu

**Affiliations:** ^1^ Consumer‐driven Grain Quality and Nutrition Center, Rice Breeding Innovations Platform International Rice Research Institute Los Baños 4030 Philippines; ^2^ Max Planck Institute of Molecular Plant Physiology Potsdam‐Golm 14476 Germany; ^3^ Center of Plant Systems Biology and Biotechnology Plovdiv 4000 Bulgaria

**Keywords:** anthocyanins, artificial intelligence, biofortification, carotenoids, genebanks, genome editing, metabolic engineering, micronutrients

## Abstract

Despite achieving substantial progress in caloric food security, micronutrient deficiencies remain a major global health challenge, highlighting a persistent disconnect between crop productivity and nutritional quality. Crop biofortification has emerged as a promising strategy to address this gap by enhancing the intrinsic nutrient content of widely consumed foods to meet dietary requirements through complementary roles of staple crops and horticultural species in improving diet quality. In this review, we reposition biofortification within a broader nutrition‐sensitive food system framework and discuss two complementary approaches to accelerate its impact. First, we examine how systematic exploration of global crop diversity can identify nutritionally superior germplasm from seed/genebanks for deployment in breeding and food systems. Second, we evaluate advances in modern breeding and genome‐editing approaches for improving minerals, vitamins, and health‐promoting phytochemicals across major crop groups. We further propose that a conserved set of nutrient‐regulatory pathways provides a unifying framework for cross‐crop biofortification, enabling more efficient, scalable strategies to enhance multiple nutritional traits to support a transition toward crop portfolios designed not only for yield and resilience but also for improved human health in the 21st century.

## Graphical abstract

This review explores how to make staple foods and horticultural crops more nutritious, including how artificial intelligence‐based screening of gene banks helps deploy nutritionally rich germplasm into breeding. Genome editing can help develop crops richer in minerals, vitamins, and health‐promoting compounds, supporting healthier diets and more resilient food systems.

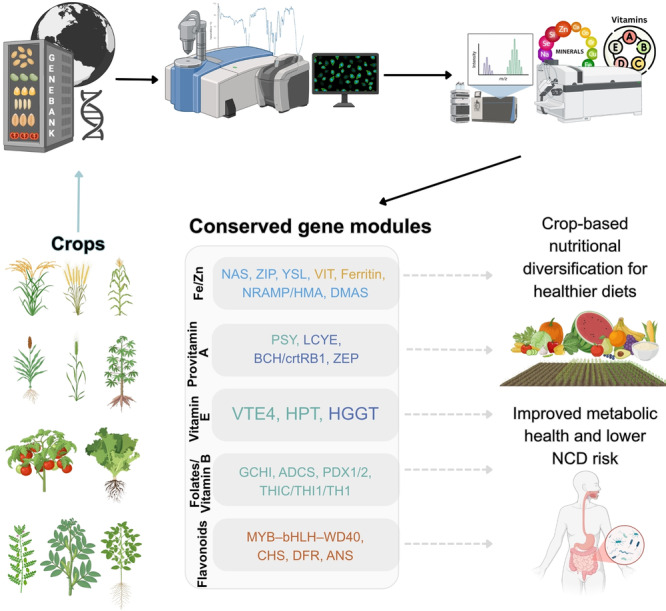

## INTRODUCTION

Despite the substantial progress achieved in addressing global food security through gains in caloric availability, malnutrition in all its forms remains persistent, revealing a disconnect between food production and nutritional quality (Meyer et al., 2012; Krug et al., 2023; Tiozon et al., 2025). Comparatively little attention has been paid to improving the intrinsic nutrient composition of the crops that anchor these diets. This neglect is particularly concerning because staple crops, including cereals, roots, and legumes, provide most of the daily calories consumed by billions of people. However, these crops are often poor sources of essential micronutrients and antioxidants, especially after post‐harvest processing. For example, milling or polishing can dramatically reduce the vitamin and mineral content of cereal grains, further weakening their nutritional contribution (Tiozon et al., 2021). This gap is intensified by the structure of global food systems, in which the most widely consumed crops contribute minimally to micronutrient and antioxidant intake.

**Figure 1:**
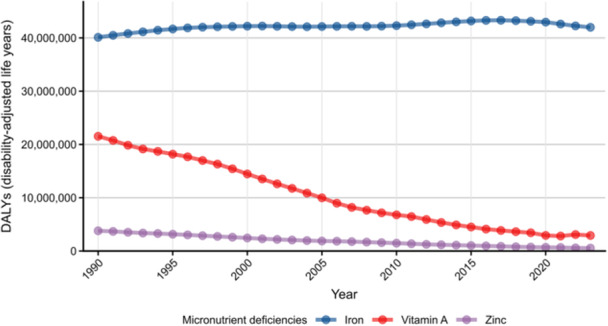
Global burden of micronutrient deficiencies Global trends in disability‐adjusted life years (DALYs) attributable to major micronutrient deficiencies from 1990 to 2023. Iron deficiency remains the dominant contributor to DALYs globally, while vitamin A and zinc deficiencies show declining but substantial burdens. These trends highlight the persistent global health impact of micronutrient malnutrition.

The result is a food system that has become increasingly efficient at delivering calories, but far less effective at delivering nutrient adequacy and chronic disease protection that persists both in high‐ and low‐income communities. These deficiencies contribute directly to anemia, impaired cognitive development, weakened immune function, poor maternal and child health, and reduced productivity, with the greatest burden borne by women and children in low‐ and middle‐income countries (Tiozon et al., 2021; Passarelli et al., 2024). Recent global modeling estimates indicate that more than five billion people consume inadequate levels of at least one essential micronutrient, with deficiencies particularly widespread for iodine, affecting about 68% of the global population, vitamin E at about 67%, calcium at about 66%, and iron at about 65% (Passarelli et al., 2024). Trends in disability‐adjusted life years (DALYs) attributable to major micronutrient deficiencies reveal a persistent but uneven pattern. For instance, iron and zinc deficiency continue to impose the largest global burden (Figure 1). In contrast, the prevalence of vitamin A deficiency has declined markedly in Western countries but remains a significant public health concern in many parts of Asia and Africa (Table 1). The burden of micronutrient deficiencies indicates that South Asia and sub‐Saharan Africa remain the epicenters of iron, zinc, and vitamin A deficiency, with disproportionately high disease burdens relative to other regions (Figure 1). These patterns closely overlap with regions characterized by heavy reliance on refined staples and ultra‐processed foods, low dietary diversity, constrained access to animal‐source foods and fortified products, and persistent poverty. Consequently, populations experience vulnerability both to micronutrient deficiency‐related disorders and to increased non‐communicable diseases (NCDs) due to high‐calorie and high‐glycemic‐index (GI) foods. Addressing these converging risks requires strategies that do not treat micronutrient deficiency prevention and chronic disease prevention as separate agendas. They also identify the very settings in which improving the intrinsic nutritional quality of staple crops could deliver the greatest public‐health benefit. This divergence suggests that current interventions have had differential success in addressing micronutrient deficiencies across nutrients, and that population‐wide improvement requires prioritizing dietary quality and food‐system transformation. Within such a framework, crop improvement is no longer merely an agricultural objective but rather becomes a component of a preventive health strategy. Aligning biofortification with nutrition‐sensitive food‐system frameworks, such as the EAT‐Lancet, offers a practical route to address both hidden hunger and the rising burden of NCDs, while also repositioning plant breeding as a central contributor to healthier and more equitable food systems.

**Table 1:** Strategic role of crop genebanks in addressing nutritional insecurity

Category	Country	Institution/indicator	City/location	Crop/resource focus	Genebank accessions	GM crops deployed already	Iron burden class	Vitamin A burden class
Global safety backup	Norway	Svalbard Global Seed Vault	Longyearbyen, Svalbard	Black‐box safety backup for crop diversity from depositors worldwide	1,301,397 samples	−	Low	Low
International CGIAR	Mexico	CIMMYT	El Batan, Texcoco	Wheat and maize, including wild relatives and breeding materials	208,698	−	High	High
International CGIAR	Lebanon	ICARDA Genebank	Terbol, Lebanon; Rabat, Morocco	Winter cereals, food legumes, forage, and rangeland species	~152,224–152,328	−	Low	Moderate
International CGIAR	Morocco	ICARDA Genebank	Terbol, Lebanon; Rabat, Morocco	Winter cereals, food legumes, forage, and rangeland species	~152,224–152,328	−	Moderate	Moderate
International CGIAR	India	ICRISAT Genebank	Patancheru/Hyderabad	Sorghum, pearl millet, and other millets, chickpea, pigeon pea, groundnut	~129,000+ official; 148,933 in Genesys	Cotton	High	High
International CGIAR	Philippines	IRRI	Los Banos	Rice and wild relatives of rice	132,876	Maize, rice, eggplant, cotton	High	High
International CGIAR	Colombia	CIAT/Alliance of Bioversity International & CIAT	Cali	Beans, cassava, tropical forages	66,531	Maize, cotton, soybean, carnation, rose, chrysanthemum, gypsophila	Moderate	Moderate
International CGIAR	Nigeria	IITA	Ibadan	Banana, cassava, yam, cowpea, soybean, maize, sorghum, Bambara groundnut, African yam bean	~31,000 overview; 38,543 in CGIAR network view	Cotton, cowpea, maize	High	High
International CGIAR	Cote d'Ivoire	Africa Rice	M'be/Bouake	Asian and African rice diversity	20,391	−	High	High
International CGIAR	Peru	CIP	Lima	Potato, sweet potato, Andean roots, and tubers	19,079	−	Moderate	Moderate
International CGIAR	Ethiopia	ILRI	Addis Ababa	Forages and some dual‐purpose crops	18,900	Cotton, maize	High	High
International CGIAR	Kenya	World Agroforestry/ICRAF	Nairobi	Agroforestry tree genetic resources, especially fruit and multipurpose trees	16,129	Cotton	Moderate	High
International specialized	Belgium	Bioversity International Musa Germplasm Transit Centre	Leuven	Banana and plantain (Musa)	1,697	−	Low	Low
Regional	Sweden	NordGen	Alnarp	Nordic agricultural and horticultural crops	~33,000–34,000	−	Low	Low
Regional	Fiji	CePaCT—Centre for Pacific Crops and Trees	Suva	Pacific taro, sweet potato, yam, banana, cassava, breadfruit, and tree species	2,600	−	Low	Moderate
Regional	Costa Rica	CATIE	Turrialba	Tropical crops and related diversity	10,950	Cotton, pineapple	Low	Moderate
National system	United States	USDA‐ARS National Plant Germplasm System (NPGS)/secure backup at NLGRP	Nationwide network; backup in Fort Collins, Colorado	Broad national crop PGRFA system across seed, clonal, and other collections	> 600,000 active accessions	Maize, soybean, cotton, canola, sugar beet, alfalfa, apple, potato, papaya, squash, pineapple		
National genebank	India	ICAR‐NBPGR National Genebank	New Delhi	Broad Indian crop genetic resources across cereals, millets, legumes, oilseeds, vegetables, etc.	> 4.68–4.7 lakh	Cotton	High	High
National genebank	China	National Genebank of China/National Crop Germplasm Preservation Center	Beijing; duplication genebank in Qinghai	Broad crop germplasm, wild relatives, in vitro and cryo holdings	> 392,000 seed accessions	Cotton, papaya, maize, soybean	High	High
National genebank	Japan	NARO Genebank	Tsukuba	Plant, plus linked animal and microorganism resources; crop PGRFA for research and breeding	> 235,000 plant accessions	−	Moderate	Moderate
National genebank	Germany	Federal Central Genebank at IPK Gatersleben	Gatersleben, with branches in Malchow and Gross Lusewitz	Agricultural and horticultural crop species, including seed, potato, and cryo collections	> 150,000 seed samples	−	Moderate	Low
National genebank	Republic of Korea	RDA Genebank/National Agrobiodiversity Center	Wanju‐gun, Jeonbuk‐do	National agrobiodiversity holdings; plant genetic resources; and associated conservation services	> 2.7 million conserved nationally	−	Moderate	Low
National genebank	Russian Federation	VIR—N.I. Vavilov All‐Russian Institute of Plant Genetic Resources	St. Petersburg	Broad world crop diversity collection	> 330,000 unique accessions; 438,951 seed accessions in low‐temperature storage	−	High	Moderate
National genebank	Canada	Plant Gene Resources of Canada (PGRC)	Saskatoon	Seed germplasm; part of the Canadian National Plant Germplasm System with clonal and potato companion sites	> 115,000 seed accessions	Canola, maize, soybean, sugar beet, alfalfa, apple, squash, cotton	Moderate	Low
National genebank	Australia	Australian Grains Genebank (AGG)	Horsham, Victoria	Cereals, pulses, oilseeds, and grain crop wild relatives	> 210,000 official; 178,265 in Genesys	Canola, cotton, banana, safflower, mustard, carnation	Low	Low
National genebank	Nigeria	NACGRAB—National Centre for Genetic Resources and Biotechnology (WIEWS NGA010)	Ibadan	Nigerian crop and wild plant germplasm	8,278	Cotton, cowpea, maize	High	High
National genebank	Israel	Israel Gene Bank for Agricultural Crops, ARO Volcani Center (WIEWS ISR002)	Beit Dagan/Volcani Center area	Agricultural crops for Israel and the region	11,802	−	Low	Low
National genebank	Philippines	NPGRL—National Plant Genetic Resources Laboratory (WIEWS PHL129)	Los Banos area	National crop genetic resources of the Philippines	4,962	Maize, rice, eggplant, cotton	High	High

Note: Target countries with deployed genetically modified (GM) crops and micronutrient‐related burden classes across selected countries and territories.

“GM crops deployed already” refers to genetically modified crops that have been commercially deployed in the corresponding country/territory. “Iron burden class” and “Vitamin A burden class” refer to the country‐level micronutrient‐related burden based on 2023 IHME DALY numbers for iron deficiency and vitamin A deficiency, respectively. Countries were classified separately for each indicator into low, moderate, or high burden using tertile‐based grouping across the included countries; low indicates the lower third of burden values, moderate the middle third, and high the upper third. Because these are DALY numbers rather than rates, they reflect absolute burden and are influenced by population size. “−” indicates that no GM crop deployment was listed for that country/territory or that burden‐class data were not provided in the input table.

Recent evidence from a randomized clinical trial further suggests that multivitamin–multimineral supplementation may modestly slow biological aging, supporting the broader concept that sustained micronutrient sufficiency contributes to healthier aging trajectories (Li et al., 2026a). Existing strategies to address micronutrient deficiencies include supplementation, industrial fortification, and dietary diversification, each of which has demonstrated important benefits but also faces practical limitations. Supplementation can deliver rapid improvements in micronutrient status but depends on sustained healthcare delivery systems, compliance, and recurring costs. Industrial fortification has achieved population‐level success in some regions, and yet, it relies on centralized processing infrastructure and may not reach rural or resource‐limited populations effectively (Tiozon et al., 2021). Dietary diversification remains the most sustainable long‐term solution but is often constrained by affordability, food availability, and cultural preferences.

Biofortification offers a complementary approach by embedding nutritional traits directly into crops, enabling repeated, cost‐effective delivery of micronutrients through already widely consumed staple foods (Li et al., 2024, 2025, 2026b; Luo et al., 2025). This intrinsic and scalable nature makes biofortification particularly well suited for reaching populations with limited access to diverse diets or fortified foods. Conventional and modern breeding efforts have already demonstrated the feasibility of this approach, with hundreds of biofortified crop varieties released globally targeted to improve the individual micronutrients, including iron‐rich beans, zinc‐enriched wheat and rice, provitamin A maize, orange‐fleshed sweet potato, and iron pearl millet (Bouis and Saltzman, 2017; Virk et al., 2021). These efforts show that agricultural improvement can be aligned with nutritional outcomes when nutrition is treated as a core breeding objective rather than a peripheral trait. Recent advances in marker‐assisted selection, genomics‐assisted breeding, genome editing, and metabolic engineering have further accelerated this shift in delivering individual micronutrient targets, but do not offer holistic solutions for addressing micronutrient deficiencies for multiple micronutrient targets. Together, these approaches now span a continuum from the identification and deployment of naturally occurring alleles to the direct redesign of metabolic pathways governing nutrient biosynthesis, transport, storage, stability, and bioavailability. They also enable the simultaneous improvement of multiple nutritional traits while maintaining yield and agronomic performance, making it increasingly realistic to embed nutrition within mainstream crop improvement pipelines.

In this review, we examine two different crop biofortification strategies to address hidden hunger. The first approach emphasizes the importance of the discovery of nutritionally valuable diversity in seed/genebanks through systematic high‐throughput phenotyping efforts for multiple nutritional targets to identify nutritionally rich grains, seeds, tubers, fruits, and vegetables. This would then be coupled to resequencing strategies to identify the target genes and rare alleles responsible for natural variation in nutritional traits and, subsequently, global redeployment of these nutritionally dense accessions to pre‐breeding or for direct deployment through the nutri‐garden concept. The second approach encompasses making rapid progress through marker‐assisted and genome‐editing precision breeding methodologies to improve multiple micronutrient targets, including vitamins, minerals, and antioxidant metabolites, in nutrition‐sensitive food systems. Finally, we consider how nutritionally enhanced crops can be positioned within broader food‐system frameworks with appropriate regulatory policies to address the food and nutritional security of Asia and Africa.

## ARTIFICIAL INTELLIGENCE‐BASED MINING OF THE GLOBAL DIVERSITY FOR MULTI‐NUTRIENT TARGETS IN THE GENEBANKS

**Figure 2:**
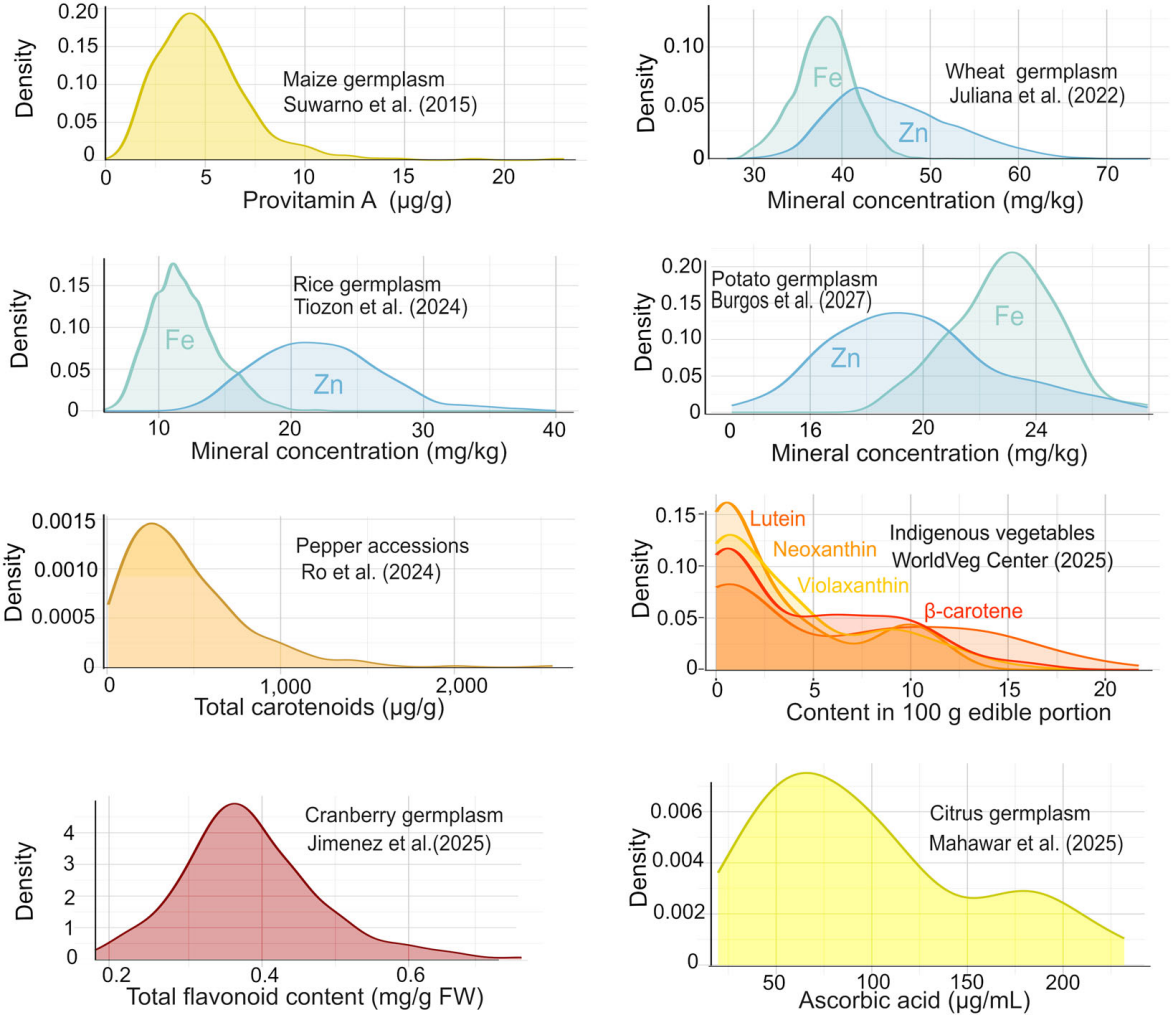
Examples of nutritional variation detected across genebank collections of major crops and horticultural species Density distributions illustrate the wide natural variation among conserved germplasm in micronutrient and bioactive compound composition, including provitamin A carotenoids in maize (CIMMYT); iron (Fe) and zinc (Zn) in wheat (CIMMYT) and rice (IRRI); minerals in potato (CIP); carotenoids in indigenous vegetables (World Vegetable Center); carotenoids in pepper (National Agrobiodiversity Center, Korea); flavonoids in cranberry (US collections); and ascorbic acid in citrus accessions from Indian genebanks. These data sets demonstrate that nutritional diversity is extensively preserved in global genetic resources and highlight the potential of genebank collections in identifying nutrient‐dense donor lines (Burgos et al., 2007; Suwarno et al., 2015; Juliana et al., 2022; Ro et al., 2024; Tiozon et al., 2024; Jimenez et al., 2025; Mahawar et al., 2025; World Vegetable Center, 2025).

Many landraces and wild relatives have evolved under distinct ecological and selective pressures, and therefore harbor chemical diversity that is not present in elite breeding lines. In this context, seed/genebanks should be viewed not merely as passive repositories of diversity, but as active starting points for nutritional innovation to mine allelic variation for elevated micronutrients, vitamins, and health‐promoting phytochemicals. Within the broader food‐as‐medicine framework, they represent a powerful, yet underutilized resource for developing staple crops for biofortification and functional health benefits (Figure 2).

**Figure 3:**
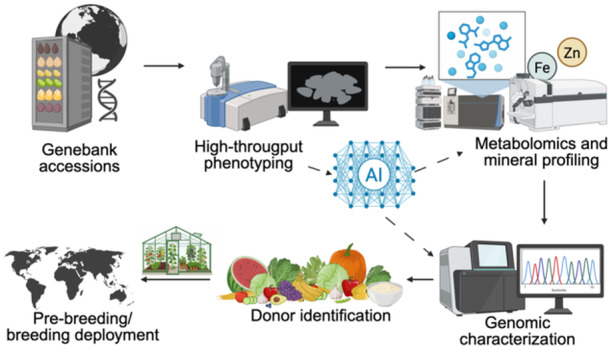
Conceptual framework for artificial intelligence (AI)‐guided discovery of nutritional traits from genebank resources Large‐scale collections are first screened using high‐throughput phenotyping technologies, including Fourier‐transform infrared spectroscopy (FTIR), near‐infrared spectroscopy (NIRS), X‐ray fluorescence (XRF), micro‐computed tomography (micro‐CT), and machine vision systems. AI models integrate these phenotypic data sets to prioritize specific subsets of accessions for metabolomic and mineral profiling. Subsequent genomic characterization allows for the identification of candidate loci and rare alleles associated with nutritional traits. Selected accessions are then advanced through donor identification and nutritional diversity panels (‘nutri‐gardens’), ultimately enabling translation into pre‐breeding and crop improvement programs.

A major emerging frontier in crop biofortification lies in combining rapid phenotyping, high‐throughput fingerprinting, and artificial intelligence (AI)‐based machine learning tools to unlock the nutritional potential already stored in 1,750 global genebanks. These genebanks conserve more than 7.4 million plant samples, representing approximately 4.9 million unique accessions from over 6,900 species, including cereals, legumes, fruits, vegetables, nuts, oilseeds, roots, tubers, and orphan crops relevant to agriculture (Bélanger and Pilling, 2019; Sreenivasulu et al., 2023). This represents a vast, yet still underexploited, source of natural variation for micronutrients, phytochemicals, protein quality, antinutrients, and traits related to digestibility. However, deployment of conventional biochemical screening to entire genebank collections would currently be impossible; hence, it is critical to identify a core collection representing the diverse collection in terms of nutritional variation through high‐throughput proxy methods (Figure 3). Emerging phenomics platforms now offer a practical alternative by enabling rapid, non‐destructive, and increasingly multiplexed assessment of grain, seed, fruit, and tissue composition through Fourier‐transform infrared (FTIR) spectroscopy, near‐infrared reflectance spectroscopy (NIRS), hyperspectral and multispectral imaging, Raman spectroscopy, X‐ray fluorescence (XRF) for mineral profiling, fluorescence imaging, thermal imaging, magnetic resonance‐based approaches, micro‐CT imaging for internal structure, and machine vision systems that capture color, morphology, texture, and other quality‐associated traits (Sreenivasulu et al., 2023; Pautong et al., 2026). Together with automated sample handling and robotics, such tools can generate compositional and structural fingerprints at a scale suitable for first‐pass screening of diverse germplasm panels to define classes and core collections. Such core collections of tens of thousands of accessions will subsequently be subjected to in‐depth biochemical characterization to survey their mineral density, vitamin and antioxidant contents, amylose structure, protein composition, untargeted metabolite analysis, or targeted assessment of antinutrient levels, albeit in a relatively low‐throughput manner. To complement such studies, AI models can be trained to associate spectral, imaging, and morphological signatures with biochemical traits, thereby enabling rapid prioritization of accessions with desirable trait combinations. In practice, the most effective machine‐learning approaches for such applications are supervised calibration models, including random forest, support vector machine, and partial least‐squares‐based methods, as well as other ensemble‐learning frameworks that integrate high‐dimensional spectral features with reference biochemical measurements. These models enable rapid and non‐destructive prediction of nutritional traits once calibrated against analytically validated training data sets. For example, Herath et al. (2024) combined ATR‐FTIR spectroscopy with machine learning to classify antioxidant polyphenols in pigmented rice with accuracies ranging from 93.5% to 100%, while also identifying spectral features associated with flavonols, flavones, and anthocyanins (Herath et al., 2024). Such approaches demonstrate that high‐throughput phenotyping, when coupled with predictive modeling, can efficiently screen large germplasm collections and capture underlying biochemical diversity.

In parallel, integrating rapid phenotyping with targeted genotyping enables associating predictive phenotypic signatures with specific loci, thereby facilitating the identification of rare or superior allelic variants (Badoni et al., 2024). This combined strategy provides an important bridge between trait prediction and allele discovery, particularly for complex nutritional traits. FTIR‐DRIFTS coupled with random forest models can accurately predict β‐carotene, α‐tocopherol, and ascorbic acid content in mango across diverse cultivars and environments, achieving high predictive performance (*R* ^2^ = 0.96–0.98) (Olale et al., 2019). These examples illustrate that AI‐assisted phenotyping is sufficiently mature to prioritize nutritionally relevant accessions across diverse germplasm panels, provided that models are rigorously validated against independent biochemical data sets and evaluated across genetic backgrounds and environments. Although prediction accuracy will vary depending on trait architecture, phenotyping platform, and population structure, spectral–machine‐learning pipelines can substantially reduce reliance on labor‐intensive wet‐chemistry screening and enable efficient identification of candidate accessions for downstream genomics, haplotype mining, and breeding.

These prioritized accessions can then be subjected to deeper multi‐omics characterization, including whole‐genome resequencing, pangenome‐guided structural variant analysis, transcriptomics, and metabolomics, to identify the regulatory networks and multigenic cascades that underlie the target genes and superior haplotypes conferring superior nutritional phenotypes. In parallel, genomic initiatives are rapidly increasing the resolution at which conserved diversity can be interrogated. Projects such as the Earth BioGenome Project (Lewin et al., 2018), the 10KP Project (Cheng et al., 2018), and efforts to sequence botanical garden collections (Zhan et al., 2026) are generating reference genomes that make it increasingly feasible to connect genebank accessions with candidate genes, metabolic pathways, and breeding‐ready haplotypes. Figure S1 illustrates the progress in genome sequencing for selected crop species, based on the NCBI Genome Database, which now includes more than 1,500 plant reference genomes of varying assembly quality. These genomic resources provide an essential bridge between conserved diversity and crop biofortification, enabling the identification of alleles that contribute not only to agronomic resilience but also to improve nutritional value (Sreenivasulu et al., 2023).

Such an approach is especially important because many biofortification traits are not controlled by single genes alone, but rather by multilayered regulatory systems involving biosynthetic enzymes, transporters, storage proteins, transcription factors, developmental regulators, and source–sink partitioning processes. Sequencing only those accessions that occupy the extremes or informative intermediate ranges of phenotypic variation can make the discovery pipeline more efficient while enriching the probability of identifying rare alleles, structural variants, favorable haplotypes, and epistatic interactions that would be missed in narrower diversity collections and/or on the evaluation of elite breeding pools alone. The most extensive international *ex situ* system is coordinated by the Consultative Group on International Agricultural Research (CGIAR), which curates about 773,000 accessions across 11 centers. Beyond conservation itself, CGIAR has also helped shape governance frameworks, such as the International Treaty on Plant Genetic Resources for Food and Agriculture (ITPGRFA) and the Nagoya Protocol, which are essential for equitable access and benefit sharing. These frameworks are highly relevant to nutritional improvement because biofortification depends on access to diverse germplasm and on the ability to mobilize useful alleles across breeding programs and geographies. AI can then be deployed to mine CGIAR genebanks, not only for phenotypic prediction but also for integrative genomic mining, by prioritizing candidate causal variants, inferring gene‐to‐trait relationships, identifying regulatory hubs, and detecting rare or lineage‐specific alleles associated with exceptional nutritional performance or resilience‐linked nutrient stability (Sreenivasulu et al., 2023). Once calibrations are defined, these high‐throughput phenotyping platforms can be applied to mine entire genebank collections for predictive fingerprinting of nutritional quality and validate the rare haplotypes through targeted haplotype mining and identify rare accessions representing multiple nutritional properties across the species. This nutritionally dense germplasm, enriched for rare alleles for multiple nutritional targets across the species, will be subjected to the nutri‐garden concept to reinfuse the genebank germplasm for immediate utilization to bring the missing nutrition to consumers in the urban environments for revitalizing the food systems, though they may not match the yield potential of improved breeding lines.

## PHYLOGENETIC ANALYSIS OF CONSERVED PATHWAYS OF MICRONUTRIENT TARGET GENES

Comparative analyses of motifs, domains, and protein sequences further reinforce the conserved‐module framework underlying crop biofortification. As illustrated in Figures S2 and S3, both *PSY* and *NAS* families retain broadly conserved core functional motifs across species, consistent with their central roles in carotenoid biosynthesis and in metal chelation, transport, and homeostasis. At the same time, the figure also highlights how gene duplication and lineage‐specific diversification have shaped these families differently, generating multiple *PSY* paralogs, and expanded *NAS* gene families in several crops, thereby enabling tissue specialization and context‐dependent sub‐functionalization (Conant and Wolfe, 2008; Van de Peer et al., 2009). In PSY, phylogenetic separation alongside conserved catalytic architecture is consistent with the recruitment of paralogs into distinct developmental and environmental roles, including carotenoid accumulation in grains or fruits, photosynthetic functions in green tissues, and stress‐responsive abscisic acid‐related functions in roots (Fraser et al., 1999; Lisboa et al., 2022). By contrast, NAS proteins show stronger conservation of the canonical NAS domain despite substantial variation in copy number, clade structure, expression domains, and, in some cases, protein length and trafficking‐related features, indicating that diversification in this family has largely occurred around a conserved biochemical function (Inoue et al., 2003; Nozoye, 2018). This pattern is especially evident in grasses, where NAS expansion aligns with the greater elaboration of endogenous Fe homeostasis and phytosiderophore‐related processes. Importantly, even small sequence changes within otherwise conserved regions can influence catalytic activity, subcellular localization, and trait expression, as shown for *PSY* alleles associated with carotenoid variation in cassava, loquat, poppy, maize, and tomato (Fraser and Bramley, 2004; Welsch et al., 2010; Shumskaya et al., 2012; Fu et al., 2014; Pollack et al., 2019). Taken together, these phylogenetic analyses underscore that *NAS* and *PSY* are not merely recurrent targets by convention, but evolutionarily conserved and functionally strategic leverage points whose successful deployment depends on both shared catalytic architecture and crop‐specific patterns of duplication, specialization, and nutrient transport biology.

## MARKER‐ASSISTED SELECTION AND GENOMICS‐ASSISTED BREEDING FOR MICRONUTRIENT BIOFORTIFICATION

Advances in high‐density genotyping, genome‐wide association studies (GWAS), quantitative trait locus (QTL) mapping, and genomic prediction have greatly expanded the ability to identify and deploy loci controlling nutrient concentration, nutrient bioavailability, and metabolite accumulation (Table 2). Rare beneficial alleles discovered in genebanks for micronutrient enrichment can be introgressed through genomics‐assisted breeding by marker‐assisted selection (MAS), pyramided through haplotype‐based selection to incorporate favorable alleles within conventional breeding pipelines. Applied across cereals, pulses, horticultural crops, nuts, and underutilized species, this framework could accelerate the identification of conserved and lineage‐specific regulatory modules for nutrient accumulation, stability, and bioavailability, thereby transforming genebanks from passive repositories into active engines of next‐generation nutritional crop improvement (Table 2).

**Table 2:** Genomics‐assisted breeding, marker discovery, and marker‐assisted selection for micronutrient, vitamin, and bioavailability traits in crops

Crop	Target nutrient/trait	Study type/breeding approach	Marker/genomic platform	Key genes/markers/QTL/loci	Population/materials	Main outcome for biofortification breeding	Reference
Amaranth (*Amaranthus hypochondriacus*)	Grain Ca, Fe, Mg, Zn	Multi‐locus GWAS	41,931 SNPs	368 significant associations, 25 environmentally stable MTAs	192 accessions across two environments	Stable MTAs and candidate genes identified for mineral‐dense amaranth breeding	(Prabhakaran et al., 2026)
Barley (*Hordeum vulgare*)	Vitamin E (tocopherols/tocotrienols)	QTL validation + haplotype analysis + marker development	KASP SNP validation, CAPS markers, sequencing	*HPT‐7H* for leaf tocopherols; *HGGT* for grain tocotrienols	Diverse barley accessions grown under field and drought conditions	A tocopherol QTL on chromosome 7H was validated across years and conditions; the Morex *HPT‐7H* haplotype was associated with higher leaf tocopherol content and higher *HPT‐7H* transcript levels; and molecular markers were developed for smart breeding of high‐vitamin‐E barley	(Schuy et al., 2019)
Cassava (*Manihot esculenta*)	Provitamin A carotenoids	KASP marker validation for MAS	KASP markers	Notably PSY2_572 for the total carotenoid content	1,677 genotypes from two breeding populations	Two KASP markers for TCC showed strong co‐segregation; PSY2_572 was particularly effective for high carotenoid selection	(Esuma et al., 2022)
Cauliflower (*Brassica oleracea* var. *botrytis*)	β‐carotene	Marker‐assisted breeding/foreground selection	Co‐dominant candidate gene markers	CFOr‐123, CFOr‐346 for the *Or* gene	Early, mid‐early, and mid‐late maturity backgrounds	Developed and validated co‐dominant markers to distinguish homozygous and heterozygous Or genotypes for rapid β‐carotene biofortification	(Singh et al., 2025)
Chickpea (*Cicer arietinum*)	Seed Fe and Zn	QTL mapping	GBS‐based linkage map	11 Fe QTL, eight Zn QTL; hotspot on *CaLG04*	F2:3 population	QTL hotspots and candidate genes identified for Fe/Zn enrichment	(Sab et al., 2020)
Common bean (*Phaseolus vulgaris*)	Seed Fe and Zn concentration/content	Meta‐QTL analysis	QTL integration across studies	12 meta‐QTL, including eight shared Fe/Zn MQTL	Meta‐analysis of 87 QTL from 7 populations	Shared MQTL identified for simultaneous Fe/Zn improvement through MAS	(Izquierdo et al., 2018)
Common bean (*Phaseolus vulgaris*)	Seed micronutrients	SNP‐QTL/GBS‐based mapping	GBS SNPs	113 significant SNPs for Mg, Mn, Fe, Ca, Cu, Zn, etc.	96 selected genotypes	Markers and candidate genes identified for nutritionally rich bean breeding	(Nazir et al., 2022)
Lentil (*Lens culinaris*)	Seed Fe and Zn concentration	GWAS/marker–trait association	1,150 SNPs	2 SNPs linked to Fe, 1 SNP linked to Zn; additional loci at lower threshold	138 cultivated accessions from 34 countries, four environments	Significant genetic variation for seed Fe and Zn; marker–trait associations and candidate genes identified for biofortification breeding	(Khazaei et al., 2017)
Maize (*Zea mays*)	Carotenoids/provitamin A	GWAS for genomics‐assisted selection	GBS SNPs, 110k SNP data set	Major loci *crtRB1*, *lcyE*, *ZEP1* plus novel loci, including auxin response factor 20, RING zinc finger, HLH DNA‐binding, and AP2‐EREBP candidates	130 diverse tropical yellow maize inbred lines	GWAS confirmed the major effects of *lcyE* and crtRB1; strongest associations were for lutein on chromosome 8 near *lcyE* and β‐carotene/β‐carotene:β‐cryptoxanthin ratio on chromosome 10 near *crtRB1*; *ZEP1* on chromosome 2 was associated with total carotenoids and zeaxanthin. Results provide a foundation for genomics‐assisted provitamin A breeding.	(Azmach et al., 2018)
Maize (*Zea mays*)	Iron homeostasis/Fe efficiency traits	GWAS/association mapping	SNPs	Genome‐wide SNPs for *NEC*, *RW*, *SDW*, *H2O*, and *SPAD* traits under Fe regimes	Association mapping population plus IBM‐derived fine‐mapping	Fe homeostasis loci identified as potential targets for MAS and functional study	(Benke et al., 2015)
Maize (*Zea mays*)	Provitamin A (β‐carotene), lysine, tryptophan	MABB/gene pyramiding	*crtRB1* 3′TE, umc1066, genome‐wide SSRs	*crtRB1*, *o2*	UMI1200β+ and UMI1230β+ crossed with HKI163	Improved lines, with β‐carotene 6.12–7.38 μg/g, lysine 0.294%–0.332%, tryptophan 0.073%–0.081%, and good agronomic performance	(Chandran et al., 2019)
Maize (*Zea mays*)	Provitamin A, lysine, tryptophan	MABB plus intercrossing	crtRB1 3′TE, umc1066, SSRs	*crtRB1*, *o2*	UMI1200/UMI1230 with CE477 and VQL1 donors	Six pyramided ICF3 lines showed 13.28–14.35 μg/g β‐carotene, 0.31%–0.34% lysine, 0.078%–0.083% tryptophan, and strong agronomic performance	(Chandrasekharan et al., 2022).
Maize (*Zea mays*)	Vitamin E, vitamin A, lysine, tryptophan	MAS/multinutrient pyramiding	Gene‐based markers + SSRs	*vte4*, *crtRB1*, *lcyE*, *o2*	Parents of four popular hybrids	Improved hybrids showed approximately 2‐fold higher α‐tocopherol, 11.48 μg/g proA, high lysine and tryptophan, and yield at par with originals	(Das et al., 2021)
Maize (*Zea mays*)	Provitamin A	Marker‐assisted pedigree breeding and MABB	InDel marker for *crtRB1*; 77 SSRs for characterization	*crtRB1* favorable allele	10 elite low‐proA inbreds crossed to donor HP702‐22; 75 hybrids evaluated	New inbreds averaged 11.01 μg/g proA and several hybrids combined > 10 t/ha yield with > 10 μg/g proA	(Duo et al., 2021)
Maize (*Zea mays*)	Vitamin E, vitamin A, lysine, tryptophan	Molecular breeding/MABB	Gene‐specific markers + SSRs	*vte4*, *crtRB1*, *o2*	Parents of APQH‐10 and APQH‐11 hybrids	Reconstituted hybrids showed 2.1‐fold α‐tocopherol, 5.3‐fold proA, 1.9‐fold lysine, and 2.0‐fold tryptophan over original hybrids with similar yield	(Hossain et al., 2023)
Maize (*Zea mays*)	Provitamin A	Marker‐assisted recurrent selection (MARS)	Marker‐assisted recurrent selection cycles	Provitamin A carotenoid loci in *HGA* and *HGB* synthetics	Two tropical synthetics and C1/C2 cycles	In HGA, β‐carotene, proA, and total carotenoids increased by 40%, 30%, and 36%, respectively, after two cycles	(Kebede et al., 2021)
Maize (*Zea mays*)	Provitamin A (β‐carotene)	Marker‐assisted backcross breeding (MABB)	crtRB1‐specific marker	Favorable 543‐bp allele of *crtRB1*	7 elite inbreds and reconstituted hybrids	β‐carotene increased from 1.4 μg/g to 8.6–17.5 μg/g in inbreds; hybrids reached 21.7 μg/g, with no yield penalty	(Muthusamy et al., 2014)
Maize (*Zea mays*)	Provitamin A (β‐carotene)	MABB	crtRB1 3′TE + 214 SSRs	*crtRB1*	UMI1200 and UMI1230 for CO6 hybrid improvement	Improved lines had 7.056–9.232 μg/g β‐carotene; hybrid ACM‐M13‐002 had 7.3‐fold higher β‐carotene, with yield comparable to CO6	(Natesan et al., 2020)
Maize (*Zea mays*)	Provitamin A, lysine, tryptophan	MABB/pyramiding	Gene‐based markers + SSR background selection	*crtRB1*, *lcyE*, *o2*	QPM inbreds QLM11–QLM14 and derived hybrids	Pyramided lines showed proA 7.14–9.63 ppm; reconstituted hybrids had 6.97–9.82 ppm proA with lysine and tryptophan enrichment and no yield penalty	(Singh et al., 2021)
Maize (*Zea mays*)	Low phytic acid (mineral bioavailability)	Marker‐assisted backcross breeding (MABB)	SSR marker	*umc2230* linked to *lpa2‐2*	Cross between low‐phytate mutant EC659418 and elite line UMI395	umc2230 co‐segregated with the low‐phytate trait and was validated for MABB of low‐phytate maize	(Sureshkumar et al., 2014)
Mungbean (*Vigna radiata*)	Grain Fe, Zn, phytic acid, tannin	GWAS	GBS‐derived SNPs	20 shared significant SNPs; 35 putative SNPs; candidate genes including Vradi07g30190, Vradi10g04830	145 diverse genotypes	Identified markers for improving micronutrients while reducing anti‐nutritional factors	(Sinha et al., 2023)
Pea (*Pisum sativum*)	Seed mineral concentrations and contents	SNP linkage mapping/QTL mapping	GBS SNPs + prior markers	46 concentration QTL and 37 content QTL	RIL population from Kiflica × Aragorn	QTL for multiple seed minerals identified as resources for MAS in pea	(Ma et al., 2017)
Pearl millet (*Pennisetum glaucum*)	Grain Fe and Zn	Association mapping	SSR + genic markers	Xpsmp2261, Xipes0180, and Xipes0096 among the strongest MTAs	130 diverse lines across three agro‐climatic zones	Favorable alleles and promising lines identified for Fe/Zn enrichment	(Anuradha et al., 2017)
Pearl millet (*Pennisetum glaucum*)	Grain Fe and Zn	Multi‐environment QTL mapping	SSR linkage map	22 QTL total; co‐mapped constitutive QTL on LG2 and LG3	F6 RIL population	Stable QTL and candidate genes identified for MAS in pearl millet biofortification	(Singhal et al., 2021)
Rice (*Oryza sativa*)	Provitamin A (Golden Rice in an elite background)	Marker‐assisted backcross introgression of transgene locus	6K SNP chip for background recovery	GR2E transgene locus	GR2E crossed into BRRI dhan29	BC5F2 lines with > 98% background recovery, 8.5–12.5 μg/g carotenoids, and yields comparable to BRRI dhan29 in multi‐location trials	(Biswas et al., 2021)
Rice (*Oryza sativa*)	Grain Fe and Zn	QTL mapping	SSR markers	qFe1.1, qFe1.2, qFe6.1, qFe6.2, qZn1.1, qZn6.2	BC2F5 population from RP‐Bio226 ×Sampada	Stable QTL and candidate genes such as OsZIP4 identified for MAS of high‐Fe/high‐Zn rice	(Dixit et al., 2019)
Rice (*Oryza sativa*)	Grain Fe and Zn	Meta‐QTL analysis	QTL integration across studies	48 MQTL across 12 chromosomes; candidate genes include *OsATM3*, *OsDMAS1*, *OsNAS1‐3*, *OsVIT2*, *OsYSL16*, *OsZIP3*, *OsZIP7*	Meta‐analysis of published QTL studies	Hotspot genomic regions identified for MAS and candidate gene prioritization	(Raza et al., 2019)
Sorghum (*Sorghum bicolor*)	Grain carotenoids/provitamin A	GWAS	GWAS SNPs	Significant SNPs, including one inside putative the ZEP ortholog	Global germplasm panel	Carotenoid variation suitable for biofortification breeding; strong evidence that ZEP is a major controller of sorghum carotenoid variation	(Cruet‐Burgos et al., 2020)
Sorghum (*Sorghum bicolor*)	Grain carotenoids/provitamin A	GWAS with MAS and GS implications	GWAS SNPs + genomic prediction	Major signal in ZEP; other carotenoid‐associated loci	446 accessions phenotyped; 345 used in GWAS; and additional 2,488 accessions used in prediction analyses	High heritability for carotenoids; the ZEP region on chromosome 6 was the most prominent association for zeaxanthin and also associated with lutein and β‐carotene; and genetic evidence supported both oligogenic and polygenic control, suggesting complementary use of MAS and genomic selection	(Cruet‐Burgos et al., 2023)
Soybean (*Glycine max*)	Vitamin E (α‐tocopherol) plus oil quality	Molecular breeding/MAS	SimpleProbe marker assay plus genotypic selection for targeted alleles	Overexpression promoter allele of *γ‐TMT3* (*VTE4*) plus *FAD2‐1A*, *FAD2‐1B*, *FAD3A*, *FAD3C*, and maturity gene *E2*	Molecular breeding scheme using PI 248397 and HOLL germplasm	Developed soybean germplasm combining elevated vitamin E with high oleic/low linolenic oil; OE *γ‐TMT3* increased α‐tocopherol and α‐tocopherol:total tocopherol ratio without compromising the high oleic trait	(Hagely et al., 2021)
Soybean (*Glycine max*)	Seed vitamin E	QTL mapping with MAS potential	SSR linkage map	Major QTL linked to Satt376, Satt286, Satt266	144 F6‐derived RILs across multiple environments	Major QTL for α‐, γ‐, δ‐tocopherol and total vitamin E identified for MAS	(Li et al., 2010)
Soybean (*Glycine max*)	Seed Fe and Zn	QTL mapping	RIL mapping + WGS support	Five QTL for Fe and five QTL for Zn	248 RILs across four environments	Major QTL and candidate genes identified to accelerate MAS for Fe/Zn soybean	(Wang et al., 2022a)
Spinach (*Spinacia oleracea*)	Leaf mineral elements (13 minerals, including Fe and Zn)	GWAS	GBS‐derived SNPs	45 strongly associated SNP markers	292 accessions from 29 countries	SNP markers and high‐mineral accessions identified for spinach nutritional breeding	(Qin et al., 2017)
Sweet corn (*Zea mays* var. *saccharata*)	Vitamin A and vitamin E	MABB/gene stacking	Gene‐specific markers	*crtRB1, vte4*, with shrunken phenotype selection for *sh2*	Parents of PSSC‐2 and ASKH‐2	Reconstituted hybrids reached 19.52 μg/g proA, with 7.8‐fold proA and 2.3‐fold α‐tocopherol improvement and yield at par with original hybrids	(Chauhan et al., 2022)
Sweet corn (*Zea mays* var. *saccharata*)	Vitamin E and folic acid	Marker‐assisted pyramiding for hybrid development	SSR diversity markers; InDel7, InDel118, SNP682	*ZmVTE4* and *ZmCTM* favorable alleles	52 inbred lines grouped into six clusters using 40 SSRs; derived F1 hybrids	Six lines carried favorable *ZmVTE4* alleles, nine carried favorable *ZmCTM* alleles, and three carried both; hybrids from genetically distant parents had superior agronomic performance; and SNP682G/G associated with higher folic acid, while *ZmVTE4* favorable alleles supported vitamin E improvement and may enhance folate stability	(Lv et al., 2022)
Wheat (*Triticum aestivum*)	Grain Mn, Fe, Cu, Zn, Se	QTL mapping + KASP validation	55K SNP array; KASP conversion	Eight QTL including co‐located QFe/Se.yaas‐2D and QMn/Zn.yaas‐4D	RIL population; validated in 149 advanced lines	Flanking SNPs converted into KASP markers and validated for MAS	(Chen et al., 2024)
Wheat (*Triticum aestivum*)	Mineral concentration under salinity	QTL mapping	Axiom Wheat Breeder's Array SNPs	49 QTL for Na exclusion and multiple mineral traits, including Fe and Zn	154 F2 lines	Mineral‐related QTL under salinity identified for stress‐resilient nutrient breeding	(Hussain et al., 2020)
Wheat (*Triticum aestivum*)	Grain Fe and Zn	GWAS	GBS markers	141 markers associated with GZnC/GFeC across data sets	5,585 CIMMYT breeding lines	Shared and environment‐consistent markers identified, but highly quantitative control suggested GS alongside MAS	(Juliana et al., 2022)
Wheat (*Triticum aestivum*)	Grain Fe, Zn, TKW	GWAS	35K Axiom Array	17 Bonferroni‐significant MTAs	280 diverse bread wheat genotypes	MTAs identified for rapid development of biofortified wheat	(Krishnappa et al., 2022)
Wheat (*Triticum aestivum*)	Grain nutrients under nitrogen treatment	High‐density SNP QTL mapping	35K Axiom array	26 QTL, including two for Fe and four for Zn	RILs from RAJ3765 × HD2329 under control and N‐deficient conditions	Nutrient QTL under contrasting N conditions identified for climate‐resilient biofortified wheat	(Malakondaiah et al., 2025)
Wheat (*Triticum aestivum*)	Grain Fe, Zn, Cd	Marker–trait association	Gene‐specific SSR markers	NAM‐B1, TaVTL5, TaVTL2, TaVTL1, HMA3, TaNRAMP3 markers	Wheat accessions grown under Zn and Cd treatments	Markers associated with Fe and Zn accumulation and low Cd accumulation, useful for MAS	(Rasheed et al., 2023)
Wheat (*Triticum aestivum*)	Grain Fe, Zn, protein, kernel traits	GWAS	35 K Axiom Array	55 MTAs, including four for Fe and two for Zn	184 diverse bread wheat genotypes	MTAs identified for grain micronutrients and quality traits; useful for MAS after validation	(Krishnappa et al., 2023)
Wheat (*Triticum aestivum*)	Grain Fe and Zn	Meta‐QTL analysis	Integrated QTL analysis	11 MQTL, including 8 shared Fe/Zn MQTL; 101 candidate genes	Meta‐analysis of prior QTL studies	Stable MQTL and candidate genes identified for MAS and biological interpretation	(Singh et al., 2022)
Wheat (*Triticum aestivum*)	Grain Zn and Fe	QTL mapping + KASP validation	QTL mapping and KASP assays	Stable Zn QTL on 3BL, 5AL, 5BL, and Fe QTL on 4BS, 4DS, 1DS, 3AS, and 6DS	245 RILs plus 125 resequenced accessions	Stable QTL and validated KASP assays identified; favorable alleles more frequent in landraces	(Sun et al., 2024)
Wheat (*Triticum aestivum*)	High grain Fe and Zn	Marker‐assisted pyramiding/forward breeding	Trait‐linked markers	*IRT2*, *MTP3*, *IREG*, *FRO7*, *YSL15*, *NAS2*	Derived lines in the PBW343 background	Pyramided lines reached 47.3–60.4 ppm Fe and 39.35–47.85 ppm Zn, with no yield penalty	(Verma et al., 2025)
Wheat (*Triticum aestivum*)	Grain Fe concentration	GWAS	660K SNP array	911 significant SNPs; stable SNPs AX‐108912427 and AX‐94729264	207 accessions across three locations	Stable Fe‐associated SNPs and candidate genes identified for Fe‐rich wheat breeding	(Wang et al., 2022b)
Peach (*Prunus persica*)	Anthocyanins/blood–flesh trait	Genetic mapping with MAS relevance	SSR linkage mapping	DBF locus; flanking SSRs AMPP157 and AMPPG178	Four successive families derived from “Wu Yue Xian”; linkage map from “D6090”	A dominant blood–flesh locus associated with anthocyanin‐rich red flesh was mapped on *LG5*, and flanking SSR markers were identified to support MAS for antioxidant‐rich peach breeding	(Shen et al., 2013)
Apple (*Malus × domestica*)	Polyphenols, including flavanols, flavonols, anthocyanins, and hydroxycinnamic acids	QTL mapping plus candidate gene mapping	Genetic maps + candidate gene markers	LAR1, HCT/HQT; 79 QTL, including stable QTL clusters	“Royal Gala” × “Braeburn” progeny	Stable QTL and candidate genes were identified for polyphenolic compounds with antioxidant properties, providing allele‐specific markers for MAS of apples with healthier phytochemical profiles	(Chagne et al., 2012)
Apple (*Malus × domestica*)	Phytochemicals, including chlorogenic acid	mGWAS plus pedigree‐based validation	~10,000 SNPs; LC‐MS and NMR metabolomics	mQTL hotspots on LG16 and LG17; chlorogenic acid mQTL on chromosome 17	124 apple genotypes, including breeding families and diverse germplasm	Multiomic integration detected and validated mQTL for health‐related phytochemicals, demonstrating a platform for phytochemical‐based molecular breeding in apple	(Bilbrey et al., 2021)
Apple (*Malus × domestica*)	Polyphenols/phenolic compounds	Untargeted mGWAS	~280,000 SNPs with UPLC‐MS metabolomics	> 630 significant loci and hotspots for flavonols, dihydrochalcones, and flavanols; candidates include bHLH and C2H2 TFs	439 diverse apple accessions	Identified major loci controlling phenolic composition and established a foundation for marker‐assisted breeding and gene editing to improve health‐beneficial polyphenols in apple	(Song et al., 2025)
Strawberry (*Fragaria × ananassa*)	Phenylpropanoids, total polyphenols, antioxidant capacity	mQTL mapping	Genetic linkage mapping/metabolite QTL analysis	QTL hotspots on LG IV‐3, V‐2, V‐4, VI‐1, and VI‐2; candidate gene *FaF3′H*	F1 population “232” × “1392” across two seasons	Stable QTL hotspots for antioxidant secondary compounds were identified, and associated markers were proposed as useful targets for MAS of high‐antioxidant strawberry cultivars	(Pott et al., 2020)
Cranberry (*Vaccinium macrocarpon*)	Anthocyanins, proanthocyanidins, acidity, Brix	QTL mapping with high‐throughput phenotyping	QTL mapping plus digital imaging phenotyping	Novel QTL for total anthocyanins, PAC, and related chemical traits	Three breeding populations with repeated ripening‐stage measurements	Novel QTL affecting anthocyanins and other health‐related traits were identified, and imaging‐based phenotyping was shown to be useful for breeding antioxidant‐rich cranberry	(Diaz‐Garcia et al., 2018)
Raspberry (*Rubus idaeus*)	Anthocyanin antioxidants	QTL mapping	Linkage mapping + HPLC phenotyping	Anthocyanin QTL on *LG1* and *LG4*; candidate regulators include *bHLH, NAM/CUC2‐like,* and *bZIP*	Progeny from *Rubus idaeus* cv. Glen Moy × *R. strigosus* cv. Latham	Stable QTL across seasons and environments were identified for anthocyanin antioxidants, supporting marker‐assisted breeding for improved nutritional and color quality	(Kassim et al., 2009)
Pineapple (*Ananas comosus*)	Flesh color/carotenoids	QTL analysis with genome‐assisted marker development	Haplotype‐resolved genome sequencing + QTL mapping	*AcCCD4* associated with white flesh and low carotenoid content	Japanese cultivar “Yugafu” and parental haplotypes	A carotenoid‐related QTL and candidate gene were identified for white flesh/low carotenoid content, providing genomic information useful for MAS in pineapple quality breeding	(Nashima et al., 2022)
Guava (*Psidium guajava*)	Vitamin C, sugars, TSS, acidity	QTL mapping	Linkage map with 195 markers	58 QTL; 13 stable QTL across environments; seven QTL clusters	Intraspecific cross “Allahabad Safeda” × Purple Guava landrace	Stable multi‐environment QTL were identified for vitamin C and fruit quality traits, providing a foundation for marker‐assisted breeding of nutritionally improved guava	(Maan et al., 2023)
Tomato (*Solanum lycopersicum*)	Fruit mineral contents	QTL mapping for molecular breeding	High‐density linkage map with co‐dominant markers	Fruit mineral QTL and epistatic QTL; flanking candidate genes	RIL population from *S. lycopersicum* × *S. pimpinellifolium* evaluated over three seasons	Fruit mineral QTL were identified and flanked by co‐dominant markers useful for MAS, enabling breeding of tomato with improved nutritional mineral composition	(Capel et al., 2017).
Tomato (*Solanum lycopersicum*)	Sugars, acids, vitamin C, carotenoids	Wide‐genome QTL mapping	Genetic map with 353 molecular markers	QTL near Sucrose Phosphate Synthase, 1‐deoxy‐D‐xylulose 5‐phosphate synthase, Tocopherol cyclase	RIL population derived from *S. pimpinellifolium* TO‐937	QTL and candidate genes for vitamins and carotenoids were identified, providing molecular tools for MAS of improved tomato nutritional quality	(Capel et al., 2015).
Eggplant (*Solanum melongena*)	Fruit anthocyanins/nonphotosensitive purple color	QTL‐seq, fine mapping, and marker validation	QTL‐seq, linkage mapping, validated KASP markers	*SmNPS10.1*, *SmMYB113*; KASP markers 21QP381 and 23QP715	Multiple populations, including NPS and photosensitive lines	A major anthocyanin‐regulating locus was fine‐mapped and KASP markers were validated for direct MAS of fruit color and anthocyanin‐associated traits in eggplant	(Xia et al., 2026)
Eggplant (*Solanum melongena*)	Anthocyanin pigmentation	High‐resolution linkage mapping/QTL mapping	GBS‐derived SNP map	Major and minor QTL for anthocyanin traits across organs	163 RILs from ‘305E40’ × ‘67/3’	High‐resolution QTL mapping identified loci and candidate genes for anthocyanin pigmentation, supplying information for marker development and MAS of pigmented eggplant	(Toppino et al., 2020)
Rapeseed (*Brassica napus*)	Seed tocopherol content and composition/vitamin E	Joint QTL mapping, GWAS, and candidate gene mapping	QTL mapping, GWAS, homologous gene mapping	33 unique QTL; 61 associated loci; candidate tocopherol biosynthesis genes	TNDH population, RC‐F2 population and 142 accessions	Major loci for tocopherol content and composition were identified, and near‐isogenic lines confirmed breeding utility, supporting MAS for high‐vitamin‐E rapeseed	(Wang et al., 2012)

Abbreviations: GBS, genotyping‐by‐sequencing; GWAS, genome‐wide association study; KASP, Kompetitive allele‐specific PCR; MABB, marker‐assisted backcross breeding; MARS, marker‐assisted recurrent selection; MAS, marker‐assisted selection; MQTL, meta‐QTL; MTAs, marker–trait associations; QTL, quantitative trait loci; RIL, recombinant inbred lines; SNP, single‐nucleotide polymorphism; SSR, simple sequence repeat.

### Biofortification to enrich grain mineral enrichment strategies

Given that most nutrient traits are controlled by multiple interacting processes, pyramiding complementary loci is increasingly favored over single‐gene selection. This is particularly evident for mineral biofortification, where uptake, xylem–phloem transport, vacuolar partitioning, remobilization, and sink storage all contribute to the final grain content. For example, wheat lines carrying favorable alleles of *iron‐regulated transporter 2* (*IRT2*), *metal tolerance protein 3* (*MTP3*), *iron‐regulated gene* (*IREG*), *ferric reductase oxidase 7* (*FRO7*), *yellow stripe‐like transporter 15* (*YSL15*), and *nicotianamine synthase 2* (*NAS2*) achieved grain iron levels of up to 60 ppm and zinc levels approaching 48 ppm without yield penalties (Verma et al., 2025). In wheat, association mapping identified markers linked to genes involved in mineral transport and homeostasis, including *NAM‐B1*, *TaVTL5*, *TaVTL2*, *TaVTL1*, *HMA3*, and *TaNRAMP3*, highlighting transport, vacuolar storage, and detoxification processes as major determinants of Fe and Zn accumulation while also contributing to reduced cadmium uptake (Rasheed et al., 2023). Most contemporary wheat varieties remain below the HarvestPlus biofortification breeding targets of 38 mg kg^−1^ for zinc and 59 mg kg^−1^ for iron, levels considered sufficient to contribute approximately 30% of human nutritional requirements (Devkota et al., 2026; Tiozon et al., 2026).

In rice, integration of multiple QTL studies identified 48 meta‐QTL associated with grain Fe and Zn, encompassing candidate genes such as *OsNAS1‐3*, *OsVIT2*, *OsDMAS1*, *OsZIP3*, *OsZIP7*, and *OsYSL16*, all of which are mechanistically linked to metal chelation, vacuolar partitioning, transport, and homeostasis (Raza et al., 2019). Additional study identified that meta‐QTL regions derived superior haplotypes associated with higher grain Zinc content (Hap1 for *OsZIP5*, *OsNRAMP7*, and *OsMT1f*; Hap2 for *OsRab6B2*, *OsDMAS1*, and *OsZIP7*; Hap3 for *OsZIP9 and OsFRO1*, and Hap4 for *OsMT1g*, respectively), derived from the candidates of regulators, genes for metal homeostasis, root exudate production, metal uptake, transport, partitioning, and loading into rice grains (Joshi et al., 2023). Likewise, based on haplotype enrichment strategies for minor alleles through genome‐wide marker–trait associations, multi‐mineral‐enriched lines for Fe, Zn, Mn, and other minerals were identified from the 3K resequencing resources (Pasion et al., 2023). Similar analysis in common bean identified 12 meta‐QTL, including several regions affecting both iron and zinc, thereby highlighting shared genomic control of multiple minerals and reinforcing the value of these genomic regions as targets for simultaneous improvement (Izquierdo et al., 2018). Meher et al. (2023) discussed genomic prediction models for micronutrient characteristics (iron, zinc, and β‐carotene) in bread wheat (Meher et al., 2023). Even when the training and test sets came from different environments, BayesRR achieved the best performance among the eight models examined. The authors recommended using the BayesRR as the best methodology for estimating genomic estimated breeding values (GEBVs) to predict micronutrient content before using them for genomic selection to increase the micronutrient content of wheat grains. For the grain's Fe and Zn concentrations, Parmar et al. (2023) found five large main‐effect QTLs for iron content and four large main‐effect QTLs for zinc content, of which three QTLs (*qFe‐Ah01*, *qZn‐Ah03*, and *qFe‐Ah11*) were responsible for increasing both iron and zinc contents in the grain (Parmar et al., 2023). Additionally, QTLs and genes involved in epistatic interactions were identified. Several candidate genes located within these QTL regions include ZRT/IRT‐like protein (ZIP) metal transporters, basic leucine zipper (bZIP) transcription factors, vacuolar iron transporter (VIT) genes, WRKY transcription factors, vacuolar protein sorting‐related proteins, late embryogenesis abundant (LEA) proteins, and basic helix–loop–helix (bHLH) transcription factors. These genes have the potential to be key players in boosting the Fe and Zn contents of groundnut seeds. In maize, marker‐assisted approaches have identified promoter variation in *ZmNAC78*, a transcription factor that regulates iron transporter expression and is associated with increased kernel iron (Yan et al., 2023), underscoring the overlap between precision and conventional genomic strategies. Comparable insights have emerged from pearl millet and chickpea, where both association mapping and linkage mapping have identified a limited number of reproducible loci for Fe and Zn. These studies indicate that mineral improvement across species often converges on recurring biological functions involving transport, chelation, remobilization, and sink loading.

One of the earliest practical demonstrations of MAS for nutritional improvement focused on reducing grain phytate. In maize, marker‐assisted backcross breeding was used to introgress the *lpa2‐2* low‐phytate allele, thereby reducing phytic acid and improving mineral availability. The simple sequence repeat (SSR) marker *umc2230* showed strong co‐segregation with the low‐phytate trait and enabled efficient selection of improved lines (Sureshkumar et al., 2014). This study illustrated an important principle that remains highly relevant, wherein breeding for nutrition is not limited to increasing nutrient concentration but can also target the biochemical context that determines nutrient absorption by reducing the levels of antinutrients.

### MAS‐based biofortification to enrich vitamins

MAS has been equally important for vitamin biofortification, particularly in carotenoid breeding. In maize, numerous programs have targeted favorable alleles of β‐carotene hydroxylase 1 (*crtRB1*) and lycopene ε‐cyclase (*lcyE*), two major branch‐point regulators of carotenoid composition. Marker‐assisted backcross breeding using a *crtRB1*‐specific marker increased β‐carotene levels from approximately 1.4 μg g^−1^ to as high as 17.5 μg g^−1^ in elite maize lines and up to 21.7 μg g^−1^ in derived hybrids, without compromising yield (Muthusamy et al., 2014). Marker‐assisted recurrent selection has also demonstrated the capacity for incremental improvement in provitamin A carotenoids, with β‐carotene increasing by up to 40% after two selection cycles in tropical maize populations (Kebede et al., 2021). Subsequent pyramiding of *crtRB1*, *lcyE*, and *opaque2* (*o2*) further enhanced nutritional quality by increasing provitamin A together with lysine and tryptophan (Chandran et al., 2019; Chandrasekharan et al., 2022). Similarly, pyramiding favorable alleles of *tocopherol methyltransferase* (*vte4*), *crtRB1*, *lcyE*, and *o2* generated hybrids with increased α‐tocopherol, provitamin A, lysine, and tryptophan while maintaining agronomic performance (Das et al., 2021; Hossain et al., 2023). In sweet corn, marker‐assisted pyramiding of favorable alleles in *ZmVTE4 and Zea mays chorismate transporter* (*ZmCTM*) improved vitamin E and folic acid while maintaining hybrid performance (Lv et al., 2022). In soybean, molecular breeding targeting the γ‐tocopherol methyltransferase (*VTE4*) pathway enabled the development of lines with elevated α‐tocopherol combined with improved oil quality (Hagely et al., 2021). Similarly, in barley, haplotype analysis and marker development for *homogentisate phytyltransferase* (*HPT‐7H*) and *homogentisate geranylgeranyl transferase* (*HGGT*), genes involved in tocopherol and tocotrienol biosynthesis, have provided useful tools for breeding high‐vitamin E varieties (Li et al., 2010; Schuy et al., 2019). Together, these studies point to a conserved set of vitamin E pathway genes that can be exploited across species using marker‐trackable alleles or haplotypes. These examples highlight the power of MAS where large‐effect alleles and operational markers are available.

In maize and sorghum, GWAS and QTL mapping have identified loci controlling carotenoid variation, including major genes such as *crtRB1*, *lcyE*, and *zeaxanthin epoxidase* (*ZEP*), which regulate key steps in carotenoid biosynthesis and degradation (Azmach et al., 2018; Cruet‐Burgos et al., 2020; Cruet‐Burgos et al., 2023). In cassava, Kompetitive allele‐specific PCR (KASP) markers linked to *phytoene synthase 2* (*PSY2*) have been validated across large breeding populations and have been shown to reliably predict the total carotenoid content, thereby facilitating efficient selection of provitamin A‐rich genotypes (Esuma et al., 2022). Similar principles are now emerging in horticultural crops. In pineapple, haplotype‐resolved genome sequencing and QTL analysis identified *carotenoid cleavage dioxygenase 4* (*AcCCD4*) as a candidate gene underlying white flesh and low carotenoid content, providing a genomic basis for marker‐assisted improvement of carotenoid‐related fruit quality (Nashima et al., 2022). Meanwhile, in cauliflower, co‐dominant markers linked to the *Orange* (Or) gene enabled discrimination of homozygous and heterozygous genotypes for rapid β‐carotene biofortification (Singh et al., 2025).

In guava, multi‐environment QTL mapping identified stable loci for vitamin C, sugars, total soluble solids, and titratable acidity, indicating that nutritional quality traits can be genetically dissected and incorporated into breeding pipelines (Maan et al., 2023). At the same time, studies of strawberry under field and maturity‐stage interactions underscore that traits such as vitamin C are strongly shaped by genotype × environment × developmental effects, meaning that marker deployment must often be coupled with multi‐environment phenotyping and careful maturity standardization (Ali and Serce, 2022). In tomato, QTL mapping using interspecific populations identified genomic regions affecting fruit mineral contents, vitamins, carotenoids, sugars, and acids, with several loci flanked by co‐dominant or candidate gene markers suitable for marker‐assisted breeding (Capel et al., 2015, 2017). In eggplant, high‐resolution mapping and QTL‐seq have resolved loci controlling anthocyanin pigmentation, including *MYB transcription factor 113* (*SmMYB113*) and associated validated KASP markers, thereby providing practical tools for selecting fruit color types enriched in anthocyanin‐associated antioxidant compounds (Toppino et al., 2020; Xia et al., 2026). These examples suggest that, while horticultural MAS has often been framed around visible quality traits, many of those traits are tightly linked to carotenoids, anthocyanins, polyphenols, vitamin C, and mineral composition, making them directly relevant to nutritional improvement.

Overall, marker‐ and genomics‐assisted breeding provide practical routes for exploiting natural variation in crop nutritional traits. Their greatest strengths lie in the identification, validation, and pyramiding of favorable alleles across conventional breeding backgrounds. However, many nutrient traits remain strongly polygenic, environmentally sensitive, or constrained by tissue‐specific metabolic architecture. In such cases, precision breeding offers an important complementary route, enabling direct manipulation of the pathways and processes that natural variation alone may not be sufficiently optimized to deliver multiple nutritional requirements.

## GENOME EDITING AND GENETIC ENGINEERING INTERVENTIONS TO ENHANCE MICRONUTRIENTS AND ANTIOXIDANTS IN CROPS

**Figure 4:**
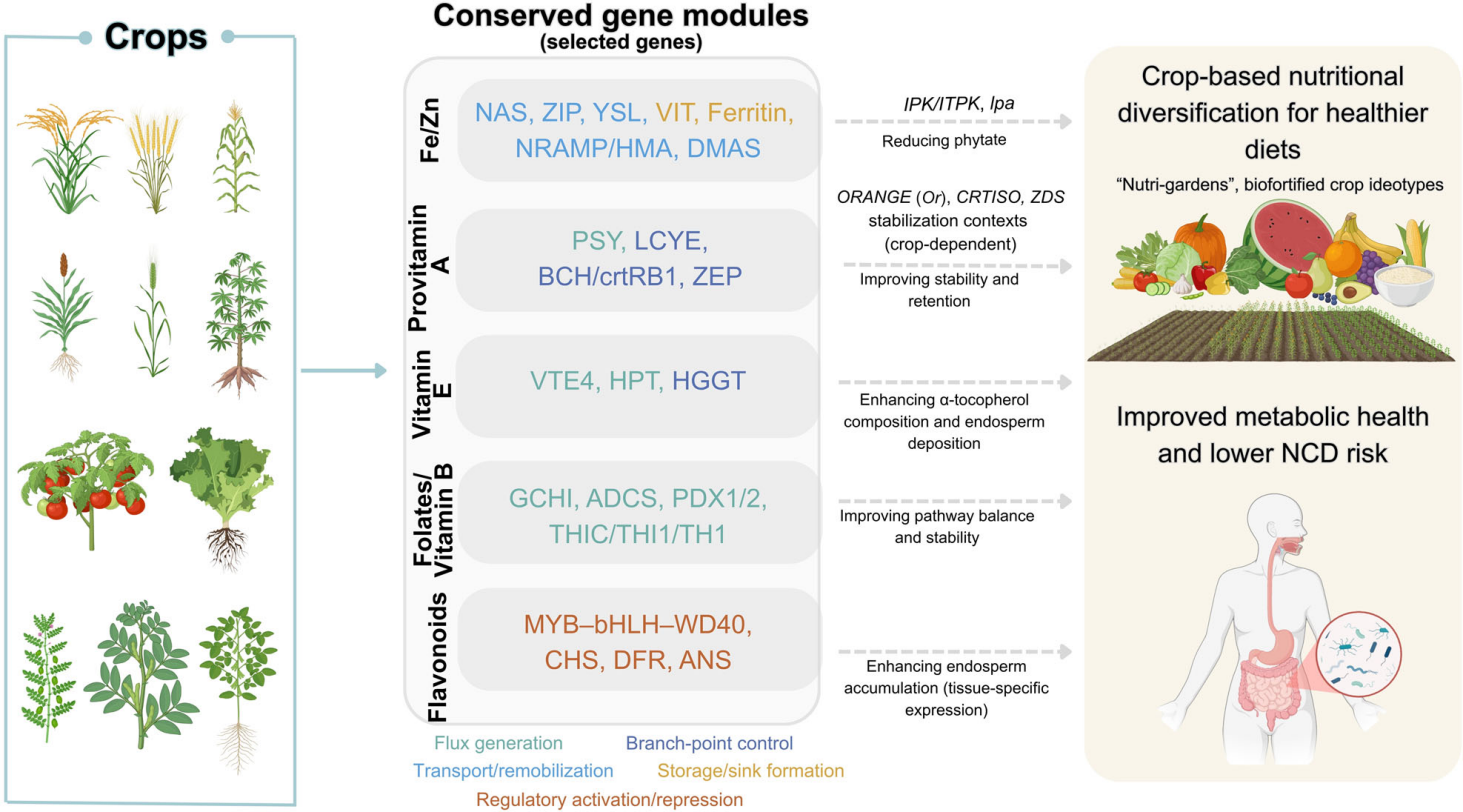
Genome editing of conserved gene modules that control multiple nutritional traits across crop species for nutritional biofortification Schematic representation of conserved genetic modules involved in the biosynthesis, transport, storage, and regulation of nutritionally important metabolites and micronutrients across diverse crop species. Major gene families associated with iron and zinc homeostasis (*NAS*, *ZIP*, *YSL*, *VIT*, *Ferritin*, *NRAMP/HMA*, and *DMAS*), provitamin A carotenoid biosynthesis (*PSY*, *LCYE*, *BCH/crtRB1*, and *ZEP*), vitamin E metabolism (*VTE4*, *HPT*, and *HGGT*), folate and vitamin B biosynthesis (*GCHI*, *ADCS*, *PDX1/2*, and *THIC/THI1/TH1*), and anthocyanin and flavonoid accumulation (MYB–bHLH–WD40 regulatory complex, CHS, DFR, and ANS) represent conserved modules that can be targeted to enhance nutritional quality. Additional pathway modifications, such as reducing phytic acid through *IPK/ITPK* and *lpa* alleles; stabilizing carotenoids via *ORANGE* (*Or*), *CRTISO*, and *ZDS* contexts; and improving vitamin retention, contribute to enhanced nutrient bioavailability and metabolic stability in crops. These gene modules operate through coordinated processes, including metabolic flux generation, branch‐point regulation, transport and remobilization, storage/sink formation, and transcriptional control. Integrating these conserved pathways across cereals, legumes, roots and tubers, and horticultural crops enables crop‐based nutritional diversification through biofortified ideotypes and “nutri‐garden” systems, ultimately contributing to improved metabolic health and a reduced risk of malnutrition and non‐communicable diseases (NCDs).

Precision breeding, including genome editing and metabolic engineering, has transformed the scope of crop nutritional improvement (Table 3). Unlike conventional breeding, which depends on recombination and phenotypic selection, precision approaches enable direct intervention in genes that control pathway flux, branch‐point partitioning, transport, storage, tissue‐specific deposition, micronutrient stability through regulating degradation pathways, and antinutrient accumulation (Figure 4). These strategies are particularly powerful when natural allelic variation is limited, or when the desired biochemical change requires coordinated rewiring of metabolic pathways. They are also increasingly relevant to mainstream crop improvement because they can complement breeding pipelines aimed at combining high nutrient density with competitive yield, resilience, and farmer‐preferred performance. Importantly, recent work shows that these approaches are no longer confined to staple cereals but are increasingly being extended to horticultural crops, including fruits and leafy vegetables. However, in these systems, most precision‐breeding efforts have so far been directed toward traits such as shelf‐life, ripening control, appearance, and disease resistance, with limited examples in the enhancement of iron, zinc, vitamins, or antioxidants. Even so, a growing number of studies now demonstrate that nutritional quality can also be targeted in horticultural crops, providing early proof of concept for the precision biofortification of fruits and vegetables (Lobato‐Gomez et al., 2021).

**Table 3:** Genome editing and metabolic engineering for nutritional biofortification

Crop	Gene	Method	Nutrient	Increase	Tissue	Validation	Reference
Banana	*LCYε*	CRISPR/Cas9	Vitamin A (β‐carotene)	~6×	Fruit	Greenhouse	(Kaur et al., 2020)
Banana	*Soybean ferritin*	Metabolic engineering	Iron; Zinc	Fe 6.32×; Zn 4.58×	Leaf	Greenhouse	(Kumar et al., 2011)
Canola	*HvHGGT*	Metabolic engineering	Vitamin E	4×	Seed	Greenhouse	(Deng et al., 2023)
Cassava	*AtDXS*; *crtB*	Metabolic engineering	Vitamin A	Increased carotenoids	Storage root	Greenhouse	(Sayre et al., 2011)
Cassava	*FEA1*	Metabolic engineering	Iron	10 → 36 ppm	Storage root	Greenhouse	(Ihemere et al., 2012)
Cassava	*AtVIT1*	Metabolic engineering	Iron	3–7×	Storage root	Field trial	(Narayanan et al., 2019)
Cassava	*AtIRT1*; *AtFER1*	Metabolic engineering	Iron; Zinc	Fe 7–18×	Storage root	Field trial	(Narayanan et al., 2019)
Cassava	*AtPDX1.1*; *AtPDX2*	Metabolic engineering	Vitamin B6	1.9–5.8×	Storage root	Field trial	(Li et al., 2015)
Common bean	*AtGCHI*	Metabolic engineering	Folate	3.3×	Seed	Greenhouse	(Ramirez Rivera et al., 2016)
Grapevine	*VvbZIP36*	CRISPR/Cas9	Anthocyanins	Increased accumulation	Leaf	Greenhouse	(Tu et al., 2022)
Kiwifruit	*GGPS*	Metabolic engineering	Carotenoids	~1.2× lutein	Fruit	Greenhouse	(Kim et al., 2010)
Kiwifruit	*PSY*	Metabolic engineering	Vitamin A (β‐carotene)	~1.3× β‐carotene	Fruit	Greenhouse	(Kim et al., 2010)
Lettuce	*LCY‐ε*; *uGGP1*; *uGGP2*; *CCD4a*	Multiplex CRISPR	Vitamin C; carotenoids	β‐carotene 2.7×; vitamin C 6.9×	Leaf	Greenhouse	(Livneh et al., 2025)
Lettuce	*GTPCHI*	Metabolic engineering	Vitamin B9	2–8×	Leaf	Greenhouse	(Nunes et al., 2009)
Maize	*HGGT*	Metabolic engineering	Vitamin E	6×	Kernel	Greenhouse	(Cahoon et al., 2003)
Maize	*psy*; *crtI*; *dhar*; *folE*	Multigene engineering	Vitamin A; Vitamin C; Vitamin B9	β‐carotene 169×	Endosperm	Greenhouse	Naqvi et al., 2009)
Orange	*Csβ‐CHX RNAi*; *CsFT*	Metabolic engineering	Vitamin A (β‐carotene)	Up to 36×	Pulp	Greenhouse	(Pons et al., 2014)
Pineapple	*PSY*; *βLYC RNAi*; *εLYC RNAi*	Metabolic engineering	Lycopene	Increased accumulation	Fruit	Commercialized	(Lobato‐Gomez et al., 2021)
Potato	*crtB*; *crtI*; *crtY*	Metabolic engineering	Vitamin A	~20× carotenoids	Tuber	Greenhouse	(Diretto et al., 2007)
Potato	*PDX‐II*	Metabolic engineering	Vitamin B6	107%–150%	Tuber	Greenhouse	(Bagri et al., 2018)
Potato	*GGP*	Metabolic engineering	Vitamin C	~2–3×	Tuber	Greenhouse	(Bulley et al., 2012)
Rice	*OsVIT2*	CRISPR/Cas9 knockout	Iron	Increased grain Fe	Grain	Greenhouse	(Che et al., 2021)
Rice	*OsITPK6*	CRISPR/Cas9	Mineral bioavailability	Phytate −10%–32%	Grain	Greenhouse	(Jiang et al., 2019
Rice	*OsPLDα1*	CRISPR/Cas9	Mineral bioavailability	Reduced phytate	Grain	Greenhouse	(Khan et al., 2019)
Rice	*OsVMT*	CRISPR/Cas9	Iron; Zinc	Fe 1.8–2.1×; Zn 1.5–1.6×	Endosperm	Field trial	(Che et al., 2019)
Rice	*ZmLc*; *ZmPl*; *SsCHS*; *SsCHI*; *SsF3H*; *SsF3′H*; *SsDFR*; *SsANS*	Multigene metabolic engineering (transgene stacking)	Anthocyanins	~1 mg g⁻¹ dry grain	Endosperm	Greenhouse	(Zhu et al., 2017)
Rice	*psy*; *crtI*	Metabolic engineering	Vitamin A	~37 μg g^−1^	Endosperm	Commercialized	(Ye et al., 2000)
Rice	*GTPCHI*; *ADCS*	Metabolic engineering	Vitamin B9	100×	Grain	Greenhouse	(Storozhenko et al., 2007)
Rice	*GTPCHI*; *ADCS*; *FBP*	Metabolic engineering	Vitamin B9	Increased stability	Grain	Greenhouse	(Blancquaert et al., 2015).
Rice	*OsNAS3*	Metabolic engineering	Iron; Zinc	Fe 2.9×; Zn 2.2×	Grain	Field trial	(Lee et al., 2009)
Rice	*AtPDX1.1*; *AtPDX2*	Metabolic engineering	Vitamin B6	Up to 28×	Seed	Greenhouse	(Mangel et al., 2019)
Rice	*THIC*; *THI1*; *TH1*	Metabolic engineering	Vitamin B1	3×	Endosperm	Greenhouse	(Strobbe et al., 2021)
Sorghum	*psy1*; *crtI*	Metabolic engineering	Vitamin A	4–8×	Grain	Greenhouse	(Lipkie et al., 2013)
Soybean	*GmIPK1*	CRISPR/Cas9	Mineral bioavailability	Reduced phytate	Seed	Greenhouse	(Song et al., 2022)
Strawberry	*GGP*	Metabolic engineering	Vitamin C	~2×	Fruit	Greenhouse	(Bulley et al., 2012)
Strawberry	*FaMYB5*	Metabolic engineering	Anthocyanins; proanthocyanidins	Increased accumulation	Fruit	Greenhouse	(Zhang et al., 2023)
Tomato	*SGR1*; *LCYE*; *BLC*; *LCYB1*; *LCYB2*	Multiplex CRISPR	Lycopene	5.1×	Fruit	Greenhouse	(Li et al., 2018)
Tomato	*SlMYB12*	CRISPR/Cas9 knockout	Flavonoids	Reduced naringenin chalcone; pink fruit phenotype	Peel	Greenhouse	(Deng et al., 2018)
Tomato	*SlSPL‐CNR*	CRISPR/Cas9	Flavonoids	Increased flavonoids	Fruit	Greenhouse	(Zhou et al., 2024)
Tomato	*SGR1*	CRISPR/Cas9 knockout	Vitamin C; carotenoids	Increased antioxidants	Fruit	Greenhouse	(Kim et al., 2024)
Tomato	*SlLCYe*; *SlBCH*	CRISPR/Cas9	Vitamin A	1.7–2.5×	Fruit	Greenhouse	(Liu et al., 2025)
Tomato	*SlGAD2*; *SlGAD3*	CRISPR/Cas9	GABA	7–15×	Fruit	Greenhouse	(Nonaka et al., 2017)
Tomato	*Delila*; *Rosea1*	Metabolic engineering	Anthocyanins	~28×	Fruit	Commercialized	(Butelli et al., 2008)
Tomato	*GTPCHI*	Metabolic engineering	Vitamin B9	~2×	Fruit	Greenhouse	(Diaz de la Garza et al., 2004)
Tomato	*GGP*	Metabolic engineering	Vitamin C	2.6–6×	Fruit	Greenhouse	(Bulley et al., 2012)
Tomato	*SlAN2*	Metabolic engineering	Anthocyanins	Increased accumulation	Fruit	Greenhouse	(Meng et al., 2015)
Watermelon	*ClLCYB*	Natural allele/breeding target	Lycopene	Increased accumulation	Flesh	Greenhouse	(Zhang et al., 2020)
Wheat	*TaIPK1.A*	CRISPR/Cas9	Mineral bioavailability	Reduced phytate	Grain	Greenhouse	(Ibrahim et al., 2022)
Wheat	*TaVIT2*	Metabolic engineering	Iron	> 2×	Endosperm	Greenhouse	(Connorton et al., 2017)
Wheat	*PDX1*	Metabolic engineering	Vitamin B6	34.96×	Grain	Greenhouse	(Mohsin et al., 2022)
Wheat	*ThMYC4E*	Metabolic engineering	Anthocyanins	Increased accumulation	Grain	Greenhouse	(Zhao et al., 2019)

### Minerals

Mineral biofortification through precision breeding has focused primarily on iron and zinc, given the global scale of their deficiencies. Mechanistically, these efforts target uptake, chelation, vacuolar sequestration, remobilization, sink loading, and antinutrient reduction. In rice, disruption of the *vacuolar iron transporter* (*OsVIT2*) increased iron accumulation in polished grains by altering partitioning within plant tissues (Che et al., 2021). Similarly, knockout of *OsVMT*, a *vacuolar transporter of mugineic acid*, enhanced iron and zinc accumulation by increasing the availability of metal‐chelating phytosiderophores involved in mineral mobilization (Che et al., 2019). These examples reflect a common strategy in mineral engineering: Redistribution can be as important as increased uptake. In rice, endosperm‐specific expression of ferritin increased iron concentration in polished grains by about two‐fold, while additional strategies such as nicotianamine overproduction and expression of *OsYSL2* further enhanced translocation and storage. Combining these components yielded lines with up to six‐fold higher iron in polished grains without yield penalties (Masuda et al., 2013). Comparable approaches have been applied in other crops. In wheat, endosperm‐specific expression of *TaVIT2* increased iron in white flour fractions by more than two‐fold while maintaining bioavailability (Connorton et al., 2017). In cassava, transgenic expression of iron assimilation and storage genes increased iron concentrations in storage roots by up to eighteen‐fold and zinc by up to ten‐fold without affecting yield (Narayanan et al., 2019). This confirms that stacking complementary functions, rather than altering single genes in isolation, is often necessary for substantial mineral enhancement.

In banana, expression of soybean ferritin increased iron and zinc accumulation in transgenic leaves by 6.32‐fold and 4.58‐fold, respectively, providing proof of concept that mineral loading can be engineered in this crop, although enhancement of the edible fruit tissue remains an important next step (Kumar et al., 2011). In addition, recent mechanistic studies in apple have identified regulators such as MdERF017 and the MPK6‐2–ERF4–FIT module that enhance tolerance to iron deficiency and promote Fe uptake responses (Wu et al., 2026). While these are not yet direct fruit biofortification examples, they provide promising molecular entry points for future precision breeding of Fe‐efficient fruit trees grown on calcareous soils.

Improving mineral content alone is not sufficient if bioavailability remains low. For this reason, reducing phytic acid has become an important complementary objective. Genome editing of phytic acid biosynthesis genes has been demonstrated in several crops. In soybean, CRISPR–Cas (clustered regularly interspaced short palindromic repeats–CRISPR‐associated protein) disruption of *inositol pentakisphosphate 2‐kinase 1* (*GmIPK1*) reduced phytic acid without impairing plant growth (Song et al., 2022). In wheat, editing *TaIPK1* reduced phytic acid and increased grain iron and zinc accumulation (Ibrahim et al., 2022). However, these interventions also reveal a key design constraint: Some phytic acid pathway genes play essential developmental roles, showing a trade‐off with yield and germination efficiency. In rice, a severe mutation in *inositol 1,3,4‐trisphosphate 5/6‐kinase 6* (*OsITPK6*) impaired growth and reproduction, demonstrating that not all low‐phytate targets are equally tractable (Jiang et al., 2019). Alternative targets such as *phospholipase D alpha 1* (*OsPLDα1*) have therefore been explored to indirectly reduce phytic acid through linked lipid‐metabolism pathways (Khan et al., 2019). Taken together, these studies show that mineral enhancement and mineral bioavailability must be treated as coupled objectives, and that target choice is critical to avoid agronomic penalties by using alternative strategies, such as chemical interventions to reduce phytic acid (Akabane et al., 2026; Tiozon et al., 2026). More broadly, the mineral biofortification literature remains more advanced in cereals and storage roots than in fruits, vegetables, and nuts, indicating a major opportunity for future precision designs that link transporter engineering, tissue‐specific sequestration, and bioavailability optimization in horticultural crops.

### Vitamins

Enhancing vitamin content has been a major objective of precision breeding because deficiencies of vitamins A, B‐complex vitamins, C, D, and E remain widespread. Among these, provitamin A carotenoids have received the greatest attention because of the global burden of vitamin A deficiency. Early metabolic engineering studies showed that carotenoid biosynthesis could be introduced into tissues that normally lack these compounds. The landmark example is Golden Rice, in which engineering the carotenoid pathway into rice endosperm enabled β‐carotene production in the edible grain (Ye et al., 2000). Subsequent optimization using a maize phytoene synthase substantially increased carotenoid accumulation, producing Golden Rice 2 with up to a 23‐fold increase in total carotenoids and preferential accumulation of β‐carotene (Paine et al., 2005). Mechanistically, this work established that storage tissues lacking endogenous carotenoid flux can be converted into provitamin A sinks through targeted pathway reconstruction.

A similar metabolic engineering logic has been applied to other staples. In potato, tuber‐specific expression of bacterial carotenoid pathway genes (*crtB*, *crtI*, and *crtY*) produced deep‐yellow tubers with about 20‐fold higher carotenoid content and up to a 3,600‐fold increase in β‐carotene (Diretto et al., 2007). Comparable strategies have also been used in cassava and sorghum to increase provitamin A in staple foods commonly consumed in sub‐Saharan Africa (Sayre et al., 2011). The BioCassava Plus initiative extended this concept further by combining carotenoid enhancement with iron and zinc targets in cassava storage roots (Sayre et al., 2011). These studies collectively demonstrated that metabolic engineering could create or amplify vitamin A sinks in non‐photosynthetic tissues that are otherwise nutritionally poor. A similar principle has also been demonstrated in fruit crops. In sweet orange, RNAi suppression of *β‐carotene hydroxylase* (*Csβ‐CHX*), combined with expression of the flowering regulator *CsFT* to accelerate reproductive development, generated “golden” oranges with up to 36‐fold higher β‐carotene in the pulp and enhanced *in vivo* antioxidant activity (Pons et al., 2014). In kiwifruit, transformation with carotenoid biosynthetic genes such as *PSY* and *geranylgeranyl diphosphate synthase* (*GGPS*) increased β‐carotene and lutein content, further showing that carotenoid flux can be elevated even in perennial fruit systems (Kim et al., 2010). Commercial translation has also begun to emerge, exemplified by Pinkglow™ pineapple, in which expression of a tangerine *PSY* together with RNAi suppression of endogenous lycopene cyclases redirected carotenoid flux toward lycopene accumulation in the flesh (Alvarez et al., 2021).

More recently, genome editing has enabled precise rerouting of existing carotenoid flux by manipulating branch‐point genes. In banana, CRISPR‐Cas9 knockout of *lcyε* redirected carotenoid flux toward the β‐branch pathway, increasing β‐carotene levels up to six‐fold in fruit pulp while reducing α‐carotene and lutein (Kaur et al., 2020). Similar Branch‐point rerouting has been reported in tomato and lettuce. In tomato, CRISPR knockout of *SlLCYe* and *β‐carotene hydroxylase* (*SlBCH*) increased β‐carotene accumulation without major penalties to fruit quality, firmness, shelf‐life, or sugar composition (Liu et al., 2025). In lettuce, editing *LCYε* increased β‐carotene and zeaxanthin, while additional editing of *CCD4a* and regulatory elements of *GDP‐L‐galactose phosphorylase 1* (*GGP1*) and *GGP2* enabled simultaneous enhancement of carotenoids and vitamin C (Livneh et al., 2025). These studies illustrate a recurring design principle: Branch‐point genes are often among the most effective leverage points for precision biofortification. In watermelon, natural variation in *ClLCYB* affects lycopene‐to‐β‐carotene conversion through differences in protein abundance, helping explain the emergence of red‐fleshed domesticated types (Zhang et al., 2020). Although this was not a genome‐editing study per se, it identifies a clear branch‐point target that could be exploited in future precision breeding of carotenoid‐rich fruit.

Genetic engineering studies have been applied to enrich folate and other B vitamins. Folate biofortification requires coordinated flux through both the pteridine and para‐aminobenzoate branches of the pathway, making it an inherently multicomponent target. Fruit‐specific overexpression of GTP cyclohydrolase I significantly increased folate content in tomato (Diaz de la Garza et al., 2004). In rice, simultaneous engineering of both folate branches achieved up to 100‐fold increases in grain folate levels (Storozhenko et al., 2007), while co‐expression of soybean *GTP cyclohydrolase I* (*GCHI*) and *aminodeoxychorismate synthase* (*ADCS*) increased folate content in maize and wheat by 4.2‐fold and 2.3‐fold, respectively (Liang et al., 2019). Folate enhancement has also been demonstrated in lettuce through expression of a codon‐optimized *gchI*, which generated lines with up to 8.5‐fold higher folate content (Nunes et al., 2009). These results reinforce the importance of pathway balance, rather than single‐gene overexpression alone, for vitamins produced through branched or compartmentalized metabolism. Vitamin B6 has likewise emerged as an attractive target because it contributes both to nutrient sufficiency and to cellular redox homeostasis. Overexpression of *pyridoxal 5′‐phosphate synthase subunit PDX1* (*PDX1*) in wheat seeds increased vitamin B6 up to 34.9‐fold without adverse morphological effects (Mohsin et al., 2022). Expression of Arabidopsis *PDX1.1* and *PDX2* in rice increased vitamin B6 in leaves, roots, and seeds (Mangel et al., 2019), while comparable strategies in cassava and potato enhanced vitamin B6 and improved tolerance to abiotic stress (Bagri et al., 2018). These studies suggest that some vitamin targets are particularly valuable because they couple nutritional enhancement with stress‐related physiological benefits. Additional B vitamins are also beginning to receive attention. In rice, metabolic engineering of thiamine biosynthesis through overexpression of *phosphomethylpyrimidine synthase* (*THIC*), *thiazole biosynthetic enzyme* (*THI1*), and *thiamine‐phosphate pyrophosphorylase* (*TH1*) increased vitamin B1 levels in the endosperm by about three‐fold (Strobbe et al., 2021; Li et al., 2025; Li et al., 2026b). In rice under salt stress, *branched‐chain amino acid aminotransferase 2* (*OsBCAT2*) has been implicated in promoting vitamin B5 synthesis by linking branched‐chain amino acid metabolism to pantothenate production (Sun et al., 2024). Even so, vitamins B2, B3, B7, and K remain comparatively underexplored biofortification targets. This likely reflects the greater metabolic complexity of many B‐vitamin pathways, their tight coupling with central metabolism, and the difficulty of achieving large, tissue‐specific gains without unintended trade‐offs.

More exploratory work has extended to elevate provitamin D. In tomato, CRISPR‐Cas9 knockout of the sterol reductase gene *Sl7DR2* caused accumulation of 7‐dehydrocholesterol, a precursor of vitamin D3 that can be photoconverted under UVB exposure, thereby creating a potential plant‐based dietary source of this nutrient without obvious penalties to growth or yield (Li et al., 2022).

Genome editing has also been used to broaden antioxidant vitamin enhancement through targeted enrichment of vitamin C and E, as well as carotenoids. Vitamin C biofortification has similarly benefited from targeting central pathway control points. Overexpression of *GGP*, a rate‐limiting enzyme in the L‐galactose pathway, significantly increased ascorbate in tomato, strawberry, and potato, demonstrating that relatively small interventions at key flux‐generating steps can strongly affect final vitamin accumulation (Bulley et al., 2012). The recent lettuce multiplex‐editing study extends this logic by showing that vitamin C enhancement can also be combined with carotenoid editing in a single design framework, underscoring the value of coordinated pathway engineering in leafy vegetables (Livneh et al., 2025). Vitamin E antioxidants have been enhanced through both conventional and precision approaches, but precision engineering has been especially informative for tocotrienol production. Overexpression of *HGGT* increased vitamin E content in maize and canola, elevating tocotrienols and tocopherols while improving oxidative stability of seed oils (Cahoon et al., 2003; Deng et al., 2023). These studies complement work on *VTE4*, *HPT*, and related pathway genes by showing that vitamin E improvement can be achieved through multiple biochemical routes, including changing the total pool size, altering the α‐tocopherol proportion, or increasing tocotrienol‐rich fractions depending on the crop context and the breeding objective. Related work in perennial species also points to broader applicability of this pathway logic. For example, *JrVTE1* from walnut increased tocopherol accumulation when heterologously expressed in fruit–tree systems, suggesting that tocopherol pathway components from tree crops may provide useful engineering modules for future vitamin E enhancement, although direct kernel biofortification in walnut itself remains to be demonstrated (Wang et al., 2015).

The systems‐level logic has motivated multi‐nutrient engineering. In maize, coordinated engineering of carotenoid, ascorbate, and folate pathways enabled simultaneous increases in provitamin A, vitamin C, and folate in endosperm (Naqvi et al., 2009). Such work is conceptually important because it moves beyond one‐nutrient‐at‐a‐time design and demonstrates the feasibility of stacking multiple micronutrient traits within a single staple crop.

### Antioxidants

Precision breeding has also become increasingly important for enhancing antioxidant metabolites, particularly flavonoids and anthocyanins. These compounds are attractive not only because of their direct nutritional and health relevance but also because they often contribute to plant stress adaptation and product quality. In tomato, CRISPR knockout of *SGR1* increased carotenoids, phenolics, flavonoids, and ascorbic acid while improving antioxidant activity and flavor‐related traits (Kim et al., 2024). Editing developmental regulators such as the APC/C‐associated factor *SlSAMBA* also altered flavonoid composition and soluble sugar levels (de Oliveira et al., 2025), illustrating that developmental and ripening genes can indirectly shape nutritional chemistry. Likewise, CRISPR disruption of *SlMYB12* has been used to generate pink‐fruited tomatoes by reducing peel flavonoid accumulation, providing a complementary example of how editing transcriptional regulators can profoundly alter antioxidant composition and fruit appearance, even when the breeding target is sensory rather than nutritional *per se* (Deng et al., 2018). Intriguingly, this gene was also found in a study on the domestication of the tomato metabolome with several metabolic shifts paralleling the increase in fruit size, whereas others were related to regional color preferences, with the Asian preference being for pink tomatoes (Zhu et al., 2018). This again highlights the power of evaluating the broad natural genetic variation available in genebanks. A comparative analysis of the domestication of the metabolome of a wide range of species, including, among others, our major cereals alongside tomato and watermelon, revealed that changes in metabolism across domestication appear to be highly species‐specific (Alseekh et al., 2021).

Among antioxidant traits, anthocyanin engineering has been especially informative because it illustrates the importance of transcriptional control. In grapevine, CRISPR‐Cas9 knockout of *VvbZIP36*, a negative regulator of anthocyanin biosynthesis, increased the accumulation of anthocyanin intermediates and end products such as cyanidin‐3‐*O*‐glucoside (Tu et al., 2022). This type of intervention demonstrates that regulatory repression can be an efficient target where pathway capacity already exists but is transcriptionally constrained. Metabolic engineering has gone a step further by reconstructing anthocyanin biosynthesis in tissues that normally do not accumulate these pigments. In tomato, expression of the snapdragon transcription factors *Delila* and *Rosea1* induced strong anthocyanin accumulation, producing purple fruits with antioxidant capacities comparable to berries (Butelli et al., 2008). In rice, coordinated expression of regulatory and structural components of the flavonoid pathway generated purple endosperm lines with substantial anthocyanin accumulation in cereal grain (Zhu et al., 2017). These examples are particularly important for crop biofortification because they show that tissue‐specific metabolite accumulation, a key translational bottleneck, can be engineered through coordinated pathway activation. Comparable regulatory strategies are also emerging in fruit crops. In strawberry, overexpression of *FaMYB5* increased anthocyanin and proanthocyanidin accumulation, and co‐expression with *B‐box domain protein 24* (*FaBBX24*) further promoted flavonoid biosynthesis in fruit tissue (Zhang et al., 2023). Similarly, in tomato, overexpression of *SlAN2* altered ripening physiology and enhanced anthocyanin accumulation, further illustrating how transcriptional regulators can simultaneously reshape pigmentation and broader fruit metabolic programs (Meng et al., 2015).

Precision breeding demonstrates that antioxidant enhancement is feasible through manipulation of both regulatory genes and core enzymes in the phenylpropanoid and carotenoid pathways. At the same time, these studies also highlight a recurring challenge: metabolic trade‐offs. In grapevine, for example, increased anthocyanin accumulation came at the expense of other phenylpropanoid compounds (Tu et al., 2022). Effective antioxidant engineering, therefore, requires more than maximizing pathway output. It must also account for pathway competition, metabolite stability, and tissue‐specific accumulation in the edible organ. This is especially relevant in fruits and vegetables, where consumer acceptance is tightly linked to visible traits such as flesh color, peel pigmentation, ripening behavior, and texture. As a result, precision breeding in horticultural crops often operates at the interface of nutrition, sensory quality, and marketability rather than nutritional enhancement alone.

Collectively, precision breeding complements marker‐assisted approaches by enabling targeted interventions where natural variation is insufficient, pathway architecture is restrictive, or tissue‐specific accumulation limits nutritional impact. Yet, even the most successful molecular designs ultimately matter only if they translate into affordable, acceptable, and health‐relevant foods within real diets and food systems.

## PRECISION BREEDING TO ENHANCE MULTIPLE MICRONUTRIENTS AND ANTIOXIDANTS IN MULTIPLE CROPS

A major emerging opportunity in crop biofortification is to move beyond single‐species trait discovery and instead exploit conserved nutrient‐regulatory modules that recur across cereals, legumes, roots and tubers, and horticultural crops. As illustrated in Figure 4, diverse malnutrition‐related traits, including grain iron and zinc, provitamin A, vitamin E, folates, and anthocyanin or flavonoid antioxidants, are often governed by a limited number of functionally conserved pathway nodes. These nodes correspond to recurring biological processes such as flux generation, branch‐point control, transport and remobilization, storage and sink formation, and regulatory activation or repression. This creates a strong conceptual basis for using genome editing as a precision tool to redesign nutrient accumulation across crops in a modular and transferable way. This is especially important for nutritional targets that are rare in breeding germplasm, strongly polygenic, developmentally constrained, or linked to undesirable agronomic traits. In this framework, the goal is not merely to edit one gene per crop, but to apply a pathway‐guided editing strategy centered on conserved modules that can be tuned in multiple crops. Multiple nutrient enrichment strategies for the simultaneous engineering of several crops require a practical roadmap that involves five steps.

First, conserved orthologs need to be identified, and pathway nodes for which strong mechanistic evidence and minimal pleiotropic risks are prioritized. Second, the desired edit type for each target must be defined, such as knockout, promoter tuning, allele conversion, or multiplex stacking. Third, one must deploy tissue‐specific, developmentally informed validation to ensure that the nutritional gain occurs in the edible organ and is retained through harvest, storage, and processing. Fourth, trade‐offs between yield, stress resilience, sensory quality, and consumer acceptance need to be identified. Although many biofortification studies have achieved substantial nutritional gains without measurable yield penalties, these trade‐offs remain an important design and deployment consideration. Several successful examples, including iron and zinc biofortification in wheat (Velu et al., 2018), Fe/Zn pyramiding in wheat (Verma et al., 2025), high iron and zinc cassava (Narayanan et al., 2019), ferritin‐ and nicotianamine‐based iron enrichment in rice (Masuda et al., 2013), and Golden Rice introgression into elite rice backgrounds (Biswas et al., 2021) and β‐carotene maize (Das et al., 2021; Duo et al., 2021), demonstrate that nutritional traits can be improved while maintaining agronomic performance. However, this outcome needs to be robustly tested in multi‐environment settings after accomplishing regulatory compliance. Documented trade‐offs arise through several mechanisms. Dilution effects can occur when selection for higher yield reduces grain mineral concentration, as reported for zinc in rice, where combined selection for both traits results in lower genetic gain than selection for either trait alone (Calayugan et al., 2021; Tiozon et al., 2024). Negative associations between β‐carotene and yield have been reported in maize and cassava (Chavez et al., 2005; Parkes et al., 2020), while a survey of 1,470 spring wheat lines in Australia revealed generally negative correlations between mineral nutrient content and yield (Joukhadar et al., 2021). In sweet potato, β‐carotene and starch compete for carbon allocation, illustrating how nutritional enhancement interacts directly with core storage metabolism (Gemenet et al., 2020). In addition, trade‐offs may arise at the breeding level when micronutrient traits are incorporated into already complex selection indices that include yield, stress tolerance, pest and disease resistance, processing quality, and consumer‐preferred traits. Taken together, these findings indicate that yield penalties are neither inevitable nor negligible, but context‐dependent outcomes shaped by the target trait, pathway biology, genetic background, and environment. Emerging genome‐editing platforms, including next‐generation prime editors with reduced indel formation and improved edit fidelity, as well as multiplex pegRNA‐based systems enabling precise and efficient insertion of large DNA fragments (Chauhan et al., 2025; Shi et al., 2026), provide opportunities to minimize unintended genomic perturbations and improve the precision of multi‐locus editing. While these advances do not eliminate intrinsic biological trade‐offs, they enhance the ability to distinguish true pleiotropic effects from technical artifacts and support more controlled design of multi‐trait biofortification strategies. Fifth, edited alleles need to be integrated into locally adapted breeding backgrounds so that nutritional gains are delivered in cultivars that farmers will grow and consumers will eat. In this view, the true novelty is not simply that genome editing can improve nutrients, but that it enables a modular, system‐level redesign of edible plant metabolism across species. Figure 4 provides the conceptual scaffold for this shift, suggesting that malnutrition cannot be addressed by isolated trait‐by‐trait interventions, but rather by editing a limited set of conserved gene modules that repeatedly control nutrient accumulation, retention, and bioavailability across crops. This opens the possibility of a new generation of precision‐bred crop portfolios designed not only for yield and resilience but also for healthier diets and improved metabolic outcomes.

### Multiple mineral enrichment strategies across crops

For iron and zinc biofortification, the most broadly conserved targets are genes involved in metal chelation, transport, vacuolar partitioning, remobilization, and sink loading, including members of the *NAS*, *ZIP*, *YSL*, *VIT*, *NRAMP/HMA*, and *DMAS* families. Precision editing could be used in at least three complementary ways. First, promoter editing could enhance the expression of positive regulators such as NAS or DMAS in tissues that determine nutrient loading into edible organs, such as endosperm, embryo, storage roots, or fleshy fruit tissues. Indeed, this approach has already been demonstrated to work for biofortification purposes. Qiu et al. (2025) most recently introduced the concept of “editing plasticity” to evaluate the potential of promoter editing to alter gene expression. As a proof of concept, they analyzed both exhaustive prediction and random‐knockout mutants within the promoter region of *ZmVTE4*, a key gene affecting α‐tocopherol (vitamin E) content in maize (Qiu et al., 2025). Intriguingly, a high degree of agreement between predicted and observed expression is observed, extending the range of natural variation and thereby allowing the creation of an optimal phenotype. Second, editing of transporter balance could redirect mineral flow toward the edible sink, for example, by weakening sequestration in non‐edible tissues or enhancing long‐distance mobilization via *YSL* or *ZIP* activities (Zulfiqar et al., 2024). Third, multiplex editing could combine improved accumulation with reduced anti‐nutrient burden, especially by disrupting or attenuating *IPK/ITPK* pathway genes or *lpa* loci to reduce phytate and improve mineral bioavailability (Ibrahim et al., 2022). This combination is especially attractive because it not only increases the total Fe and Zn concentrations but also reduces chelating substrates, thereby increasing bioavailability.

### Multi‐vitamin enrichment strategies across crops

For provitamin A biofortification, the most strategic intervention points lie at the level of flux into the carotenoid pathway, branch‐point partitioning, and carotenoid degradation or stabilization. Across crops, conserved nodes include PSY, LCYE, BCH/crtRB1, and ZEP, together with crop‐specific opportunities involving storage plastid development and carotenoid sequestration (Figure S3). Precision editing can therefore be designed around three principles. One is to increase precursor flux by promoting PSY activity or expression. Another is to favor the provitamin A branch by weakening competing routes, such as LCYE, or modifying hydroxylation steps, such as BCH/crtRB1. A third is to enhance carotenoid retention by editing degradation pathways or stabilization contexts, for example, through genes analogous to *Or* regulators of chromoplast formation and carotenoid packaging (Yazdani et al., 2019). Importantly, this pathway is highly sensitive to tissue context and post‐synthetic stability, so effective editing strategies should combine biosynthetic enhancement with improved sequestration and reduced turnover. This is particularly relevant in crops where carotenoids are vulnerable to oxidation during maturation, storage, or cooking.

For vitamin E enhancement, the clearest conserved editing targets include *VTE4*, *HPT*, and *HGGT* (Figure 4), which influence tocopherol and tocotrienol biosynthesis as well as α‐tocopherol enrichment. Here, genome editing offers a route to improve both total vitamin E and vitamer composition. Promoter tuning or *cis*‐regulatory editing of *VTE4* could shift flux toward α‐tocopherol, the most nutritionally relevant form, while editing *HPT* and *HGGT* could enhance accumulation in leaves, grains, and oil‐rich tissues depending on crop biology (Cahoon et al., 2003; Deng et al., 2023). These targets are particularly attractive because vitamin E improvement may also contribute to oxidative stability of lipids and shelf‐life, thereby creating a dual nutritional and post‐harvest benefit.

For folates and vitamin B‐related pathways, conserved gene nodes such as *GCHI*, *ADCS*, *PDX1/2*, and *THIC/THI1/TH1* represent opportunities to rebalance pathway flux and reduce metabolic bottlenecks. In contrast to traits governed by a single major branch‐point gene, folate accumulation often depends on coordinated precursor supply and pathway balance across subcellular compartments. Precision editing is therefore likely to be most effective when used in a stacked or multiplex format, for example, by simultaneously enhancing pterin and *p*‐aminobenzoate branches while stabilizing downstream assembly. This is a strong case for CRISPR‐based multiplex editing because the phenotype depends less on one switch and more on coordinated adjustment of several moderate‐effect nodes.

### Strategies to elevate antioxidants across crops

For anthocyanins and flavonoid antioxidants, the most transferable genome‐editing targets are not only structural genes such as *CHS*, *DFR*, and *ANS* but also the upstream transcriptional regulators of the *MYB–bHLH–WD40* module. This module is especially powerful because it acts as a regulatory hub controlling tissue‐specific activation of antioxidant pathways. In pigmented cereals, legumes, fruits, and vegetables, editing of regulatory sequences or removal of transcriptional repression could increase flavonoid accumulation in edible tissues without constitutive whole‐plant overexpression. This matters because antioxidant biofortification is often limited not by the absence of biosynthetic genes but by the lack of expression in the right tissue, developmental stage, or environmental context. Targeted promoter editing, enhancer engineering, or knockout of repressors may therefore be more desirable than simple coding‐sequence modification.

### Multiple micronutrient enrichment strategies across crops

A key strategic advantage of the conserved‐module framework is that it supports cross‐crop translation. Rather than rediscovering entirely new solutions in every species, one can identify orthologous or functionally equivalent genes that govern the same nutritional process in rice, wheat, maize, cassava, barley, tomato, eggplant, lettuce, sweet potato, banana, or legumes. This does not mean that the same edit will work identically in every crop. On the contrary, the value of the framework lies in distinguishing conserved core logic from crop‐specific execution. For example, Fe/Zn loading may always require transport, chelation, and sink deposition, but the optimal edit may differ between cereal endosperm, legume cotyledon, cassava storage root, and tomato pericarp. Likewise, provitamin A editing in maize endosperm, cassava storage roots, and cauliflower curds must account for distinct plastid types, turnover dynamics, and storage environments. Thus, conserved modules should guide target selection, while crop anatomy, tissue specificity, developmental timing, and product stability should guide the final edit design. Another important implication is that future biofortification should increasingly rely on multiplex editing and trait stacking. To systematically address malnutrition, transformation of healthier diets requires simultaneous improvement in minerals, vitamins, bioactive antioxidants, and nutrient bioavailability. Figure S3 captures this logic well: The same crop can, in principle, be edited across several conserved modules to produce nutritionally superior ideotypes. For example, a cereal could combine elevated Fe/Zn via enhanced *NAS‐YSL‐VIT* circuitry, reduced phytate via *IPK/ITPK* editing, and increased provitamin A or vitamin E via carotenoid or tocopherol pathway optimization. Likewise, vegetables and fruits could be edited to enhance carotenoid levels, vitamin C‐related pathway efficiency, anthocyanin accumulation, and mineral density. Such stacked editing strategies are especially relevant for crop‐based nutritional diversification, where a portfolio of complementary nutrient‐rich staples and horticultural crops may offer greater public health impact than any single biofortified crop alone. This framework also argues for moving beyond conventional knockout logic. For many nutrition traits, the best edits may be quantitative rather than null, because excessive perturbation can compromise fitness, yield, color, palatability, or stress responses. Therefore, the most promising precision strategies may include cis‐regulatory editing, promoter tweaking or enhancement, allele replacement, editing of upstream open reading frames, engineering of transcription factor binding sites, and base editing of catalytic or regulatory residues. These approaches allow graded tuning of pathway activity rather than binary on–off outcomes. That is particularly important when editing transporters, hormone‐connected branches, or central metabolic enzymes that also affect growth.

## NUTRITIONAL CONTRIBUTION OF ENGINEERED CROPS TO ADULT MICRONUTRIENT REQUIREMENTS AND HEALTH‐RELATED ANTIOXIDANT INTAKE THRESHOLDS

**Figure 5:**
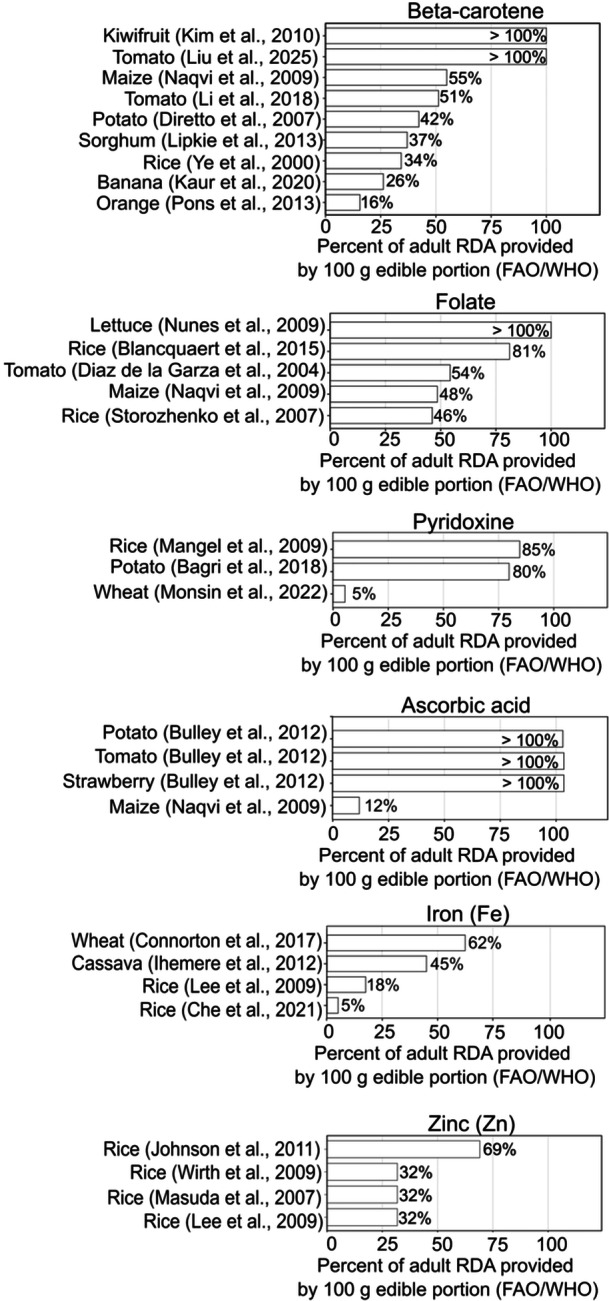
Effect of metabolically engineered crops on micronutrient requirements Percentage of the adult recommended dietary allowance (RDA) provided by a 100 g edible portion of genetically engineered crops enriched in essential micronutrients, including ascorbic acid (vitamin C), iron (Fe), folate (vitamin B9), pyridoxine equivalents (vitamin B6), thiamine (vitamin B1), zinc (Zn), and β‐carotene (provitamin A). Percentages are calculated relative to adult RDA values based on FAO/WHO dietary reference intakes (World Health Organization, 2004). Values represent nutrient contributions reported for representative engineered lines in the literature.

To evaluate the potential nutritional significance of engineered crop lines, nutrient concentrations can be interpreted relative to dietary reference benchmarks. Evaluation using recommended dietary allowances (RDAs) highlights important contrasts between staple cereals and horticultural crops in their potential nutritional impact (Figure 5). Biofortified cereals—including wheat and rice—often deliver moderate but nutritionally meaningful proportions of daily micronutrient requirements. For example, iron‐enhanced wheat provides approximately 62.5% of the adult RDA per 100 g serving, while engineered cassava provides 45% and engineered rice lines provide between 5% and 17.5% of the RDA (Lee et al., 2009; Ihemere et al., 2012; Connorton et al., 2017; Che et al., 2021). Zinc‐enhanced rice lines similarly contribute 31.8%–69.1% of the adult RDA, demonstrating the potential of improving mineral density in cereals that dominate global calorie supply (Masuda et al., 2008; Lee et al., 2009). In contrast, several horticultural crops show substantially higher vitamin concentrations relative to RDAs. For instance, provitamin A engineering through β‐carotene accumulation provides more than 50% of the adult RDA in maize and tomato, while vitamin C enrichment in potato, tomato, and strawberry can exceed 100% of the RDA per serving (Naqvi et al., 2009; Bulley et al., 2012; Li et al., 2018). Folate biofortification similarly shows strong potential, with engineered rice and maize delivering 46%–81% of the adult RDA, and lettuce lines exceeding this threshold several‐fold (Storozhenko et al., 2007; Blancquaert et al., 2015; Ramirez Rivera et al., 2016). However, the nutritional significance of these improvements must be interpreted in the context of dietary consumption patterns and food‐system scale. Cereals such as rice, wheat, and maize are consumed in large quantities globally and therefore offer a highly scalable platform for delivering micronutrients to large populations (Tiozon and Sreenivasulu, 2025). In contrast, many horticultural crops that achieve very high vitamin concentrations are consumed in smaller quantities or within more fragmented value chains, which can limit their population‐level nutritional impact despite their high nutrient density. *Ex‐ante* modeling studies of biofortification consistently emphasize the importance of targeting widely consumed staple foods to maximize public health benefits. For example, simulations evaluating iron, zinc, and vitamin A biofortification estimate that these interventions can substantially reduce the prevalence of inadequate micronutrient intake and generate highly favorable cost‐effectiveness outcomes (Lividini et al., 2018). These findings underscore that even moderate improvements in micronutrient density in staple crops can translate into substantial population‐level health gains when applied at scale.

For essential micronutrients, contributions are typically assessed against adult RDAs, whereas bioactive phytochemicals such as anthocyanins lack formal reference intakes and are therefore evaluated against intake levels associated with measurable health outcomes (Figure 5; Tables S1–S3). Human intervention and epidemiological studies suggest that anthocyanin intakes of approximately 20–40 mg day^−1^ can improve endothelial function and vascular reactivity (Figure S4), while cohort studies associate intakes of roughly 22 mg day^−1^ with reduced risk of type 2 diabetes (Guo et al., 2016; Wallace et al., 2016; Mozos et al., 2021). Higher intake ranges of 200–400 mg day^−1^ have been linked to improvements in cardiometabolic markers, including lipid profiles and insulin sensitivity, and intakes exceeding 300 mg day^−1^ have been associated with reductions in inflammatory biomarkers such as CRP and IL‐6 (Fallah et al., 2020; Sandoval‐Ramirez et al., 2022). These thresholds provide a useful framework for evaluating the potential dietary contribution of anthocyanin‐enriched crops.

Within this framework, several engineered crops reach intake levels associated with functional vascular benefits. Anthocyanin‐enhanced rice provides approximately 100 mg per 100 g serving, exceeding the lower functional thresholds and contributing roughly 50% of the cardiometabolic intake benchmark of 200 mg day^−1^ (Zhu et al., 2017). Engineered wheat and grapevine leaves provide approximately 50 and 35.9 mg per serving, respectively, also surpassing the intake range associated with vascular benefits (Zhao et al., 2019; Tu et al., 2022). Anthocyanin‐enriched tomato provides approximately 28.3 mg per serving, similarly exceeding the lower intake thresholds, whereas the engineered strawberry line contributes only 0.8 mg, illustrating that the nutritional relevance of antioxidant engineering depends strongly on the magnitude of metabolite accumulation (Butelli et al., 2008; Zhang et al., 2023). Although most engineered crops examined here do not reach the higher intake ranges associated with cardiometabolic or anti‐inflammatory benefits, they could still meaningfully contribute to the total daily anthocyanin intake when integrated into diverse diets.

### Biofortification is at the forefront of connecting nutrition‐sensitive food systems

Future perspectives to translate crop biofortification into nutrition‐sensitive food systems are the crucial need of the hour. More people need to be fed from less arable land in the face of climate change. Moreover, the need to address hidden hunger is becoming ever more apparent. Global efforts to transform food systems have increasingly emphasized the need to align human health with environmental sustainability. Frameworks such as the EAT‐Lancet Commission have been particularly influential in positioning food systems at the nexus of health, environment, climate, and social justice, while underscoring that food systems are a major driver of planetary boundary transgressions and a critical lever for improving human well‐being. However, despite their importance in shaping dietary discourse, such frameworks have largely focused on food consumption patterns, dietary shifts, and sustainability targets, implicitly treating the nutritional quality of crops as fixed, and limited efforts are made to address nutritional enrichment in staples. The nutritional quality of diets is fundamentally constrained by the composition of the crops that underpin them. In many low‐ and middle‐income countries, diets remain heavily dependent on a narrow range of staple crops that are intrinsically low in essential micronutrients and bioactive compounds. As a result, even under improved dietary scenarios, the ability to meet micronutrient and antioxidant requirements remains restricted if the major food sources themselves are nutritionally diluted. Food‐system transformation, therefore, cannot rely only on diversification, supplementation, or consumer‐level interventions. It must also address the biological quality of staple crops at the point of production.

Biofortification and precision breeding provide a powerful upstream solution to this challenge by embedding nutritional traits directly into widely consumed crops. Integrating crop nutritional enhancement into food‐system frameworks requires a shift from viewing agriculture primarily as a source of calories to recognizing it as a determinant of population health. In this context, biofortified crops can be conceptualized as nutritional carriers that deliver essential micronutrients and health‐promoting compounds at scale. Increasing iron and zinc in cereals can directly address deficiencies that affect billions of people, while enhancing antioxidants such as carotenoids and flavonoids can potentially contribute toward reducing the burden of chronic diseases linked to oxidative stress and inflammation.

The translational relevance of this approach is supported not only by biological feasibility but also by public‐health economics. Ex ante analyses indicate that biofortification is, in many contexts, a highly cost‐effective intervention for reducing micronutrient inadequacy (Lividini et al., 2018). A synthesis of 30 *ex ante* studies concluded that 96% of optimistic cost‐effectiveness estimates fell below commonly used thresholds for high cost‐effectiveness. Median optimistic cost‐per‐DALY‐saved estimates were $2.8 for iron, $9.4 for vitamin A, $42.5 for zinc, and $4.0 for multiple‐micronutrient interventions. Even pessimistic estimates remained favorable in many scenarios. These values suggest that crop biofortification can generate meaningful health returns at relatively low cost, especially where staple foods dominate the diet and delivery through conventional health systems is difficult.

Taken together, these comparisons highlight complementary roles for different crop categories within nutrition‐sensitive food systems. Biofortified cereals offer a scalable pathway to address widespread micronutrient deficiencies, given their dominant role in global diets, whereas fruits and vegetables offer opportunities to deliver high concentrations of vitamins and antioxidant metabolites that contribute to dietary diversity and long‐term health. Integrating both strategies—improving nutrient density in staple crops while strengthening value chains for nutrient‐rich horticultural foods—may therefore represent the most effective approach for bridging the gap between current dietary patterns and nutrient intake levels associated with optimal health.

## FUTURE PERSPECTIVES

Advances in genomics‐assisted breeding and precision biotechnology now make it increasingly possible to improve multiple traits within the same crop, including vitamins, minerals, antioxidant metabolites, and reduced antinutritional factors. This convergence creates an important bridge between genotype‐level intervention and population‐level health impact. Rather than treating crop breeding and public health nutrition as separate domains, biofortification makes it possible to connect them directly. A fully integrated food‐system strategy should therefore include three linked dimensions: First, conservation and utilization of genetic diversity to identify nutritionally superior alleles; second, deployment of marker‐ and genomics‐assisted, and precision breeding tools to enhance nutrient density and bioavailability in staple crops; and third, alignment with dietary frameworks that promote healthy, culturally appropriate, and environmentally sustainable consumption patterns. When nutrition is embedded directly into crop genomes, improvements in agricultural productivity can be translated more reliably into improvements in human health. The interventions that we discussed in the scope of the present review of (i) AI‐based machine learning methods to mine genebanks to identify rare accessions with multiple micronutrients in staples and horticulture crops and connecting them to nutri‐gardens and (ii) optimizing alleles of conserved network of target genes to enrich minerals, vitamins, and antioxidants to enrich the nutritional density across multiple crops to engineer biofortification are needed to revitalize the food systems of the 21st century. These novel strategies need to be coupled with population‐level bioavailability evidence in urban and rural areas to tackle the triple burden of nutritional challenges.

Despite these advances, the successful translation of biofortified crops from laboratory to field and ultimately to consumer diets depends on overcoming several non‐technical barriers. Regulatory approval processes for genome‐edited and biofortified crops remain heterogeneous across countries and can delay deployment, particularly where policy frameworks are still evolving. In this context, regulatory distinctions among genome‐editing categories (e.g., SDN1 and SDN2 versus SDN3) are increasingly important, as some jurisdictions apply less stringent oversight to edits that do not involve the integration of foreign DNA. In contrast, others maintain more uniform or precautionary regulatory frameworks. Such variability creates uncertainty in product development timelines and cross‐border commercialization pathways. In addition, consumer acceptance is influenced by perceptions of nutritional value, safety, and cultural preferences, which can vary widely across regions. Farmer adoption also depends on agronomic performance, seed availability, market incentives, and compatibility with existing production systems. Addressing these challenges will require coordinated efforts involving policymakers, breeders, industry stakeholders, and communities to ensure that biofortified crops are not only scientifically robust but also socially acceptable, economically viable, and scalable within diverse food systems.

## CONFLICTS OF INTEREST

The authors declare no conflicts of interest.

## AUTHOR CONTRIBUTIONS

R.N.T. conceptualized the study, conducted the investigation, performed formal analysis, and prepared the figures. R.N.T. and N.S. wrote the original draft of the manuscript. N.S. and A.R.F. contributed to the conceptualization of the study, supervised the project, and revised the manuscript. N.S. and A.R.F. also managed project administration and secured funding. All authors reviewed and approved the final manuscript.

## Supporting information

Additional Supporting Information may be found online in the supporting information tab for this article: http://onlinelibrary.wiley.com/doi/10.1111/jipb.70305/suppinfo

**Figure S1.** Evolution of plant genome assembly quality from 2000 to 2023
**Figure S2.** Phylogenetic relationships, conserved motifs, and domain organization of NAS and PSY proteins across plant species
**Figure S3.** Evolutionary conservation, gene family expansion, and micronutrient‐related regulatory modules across plant species
**Figure S4.** Estimated contribution of anthocyanin‐enriched crop lines to intake levels associated with human health benefits
**Table S1.** Nutritional contribution of biofortified crops relative to typical dietary intake and adult recommended dietary allowances (RDAs)
**Table S2.** Dietary anthocyanin intake thresholds associated with vascular, metabolic, and anti‐inflammatory health outcomes
**Table S3.** Estimated anthocyanin intake from biofortified crops relative to reported dietary thresholds for health benefits
