# Supplementary material for: Reconfiguring biofortification strategies to transform food systems and address micronutrient deficiency of the 21st century
Source: J Integr Plant Biol. 2026 Jun 8;68(8):2511–47. doi: 10.1111/jipb.70305 (PMC13446593; doi:10.1111/jipb.70305)
Supplement: Supplementary file 1 [file JIPB-68-2511-s001.docx]

**Supplementary Data**

**Figure S1. Evolution of plant genome assembly quality from 2000 to 2023.**

The number of plant genomes sequenced per year categorized by assembly level, including contig-, scaffold-, and chromosome-level assemblies. Early genome sequencing efforts were dominated by fragmented contig and scaffold assemblies, reflecting technological limitations in sequencing and assembly methods. Over time, advances in long-read sequencing technologies, improved assembly algorithms, and chromosome-conformation capture approaches have enabled the generation of increasingly complete chromosome-level assemblies. This transition highlights the rapid improvement in plant genome resources over the past two decades, providing higher-resolution genomic frameworks that facilitate gene discovery, comparative genomics, and crop improvement research.

**Figure S2. Phylogenetic relationships, conserved motifs, and domain organization of NAS and PSY proteins across plant species.**

**(A)** Phylogenetic tree, conserved motif distribution, protein length, and conserved domain architecture of nicotianamine synthase-like proteins across representative monocot and dicot species. Motifs 1–10 are indicated by different colors, and conserved domains are shown on the right. Dicot and monocot sequences are distinguished by orange and green labels, respectively. **(B)** Phylogenetic tree, conserved motif distribution, protein length, and conserved domain architecture of phytoene synthase-like proteins across representative monocot and dicot species. Motifs 1–10 are indicated by different colors, and conserved domains are shown on the right. Protein length is shown in amino acids. Dicot and monocot sequences are distinguished by orange and green labels, respectively.

**Figure S3. Evolutionary conservation, gene family expansion, and micronutrient-related regulatory modules across plant species.**

****(A)** Gene duplication patterns of the nicotianamine synthase (NAS) gene family across representative monocot and dicot genomes. The stacked bar plots show the number of NAS genes classified according to duplication mode, including no duplication, tandem duplication, segmental or block duplication, and combined tandem and block duplication events. **(B)** Gene duplication patterns of the phytoene synthase (PSY) gene family across representative plant genomes. The distribution of duplication modes highlights lineage-specific expansion and diversification of carotenoid biosynthesis-related genes across crop and model plant species. **(C)** Comparative presence–absence matrix of genes and pathways associated with iron uptake, micronutrient transport, and nutritional homeostasis. The heatmap summarizes the distribution of clade identity, iron uptake strategy, NAS, NAAT/DMAS, YSL family, ZIP family, VIT/VTL transporters, ferritin, and PSY across major plant species. Color annotations distinguish dicots and monocots, Strategy I and Strategy II iron uptake systems, combined strategies, and gene presence or absence. The figure highlights evolutionary differences in iron acquisition mechanisms and the conservation or diversification of nutrient regulatory modules across plant genomes.**

**Figure S4. Estimated contribution of anthocyanin-enriched crop lines to intake levels associated with human health benefits.**

Percentages represent the proportion of four intake thresholds associated with specific health outcomes based on a typical 100 g edible portion: vascular function (20 mg day⁻¹), reduced risk of type 2 diabetes (22 mg day⁻¹), cardiometabolic traits (200 mg day⁻¹), and reduction of inflammatory biomarkers (300 mg day⁻¹). Bars indicate the percentage of each intake threshold achieved by the reported anthocyanin content of the engineered crop lines.

| **Table S1. Nutritional contribution of biofortified crops relative to typical dietary intake and adult recommended dietary allowances (RDA)** | | | | | | | |
| --- | --- | --- | --- | --- | --- | --- | --- |
| **Crop** | **Nutrient** | **Analyte** | **µmol g⁻¹** | **Usual consumption** | **Amount per usual consumption (µg)** | **% adult RDA met** | **Author** |
| Cassava | Iron | Fe | 0.645 | 100 g edible portion | 3,600 | 45.00% | Ihemere et al., 2012 |
| Rice | Iron | Fe | 0.0716 | 100 g edible portion | 400 | 5.00% | Che et al., 2021 |
| Rice | Iron | Fe | 0.2507 | 100 g edible portion | 1,400 | 17.50% | Lee et al., 2009 |
| Wheat | Iron | Fe | 0.895 | 100 g edible portion | 5,000 | 62.50% | Connorton et al., 2017 |
| Banana | Vitamin A | β-carotene | 0.0447 | 1 medium fruit (~118 g) | 2,832 | 26.20% | Kaur et al., 2020 |
| Kiwifruit | Vitamin A | β-carotene | 7.998 | 1 medium fruit (~76 g) | 326,420 | 3022.40% | Kim et al., 2010 |
| Maize | Vitamin A | β-carotene | 0.1104 | 100 g edible portion | 5,932 | 54.90% | Naqvi et al., 2009 |
| Orange | Vitamin A | β-carotene | 0.0224 | 1 medium fruit (~140 g edible portion) | 1,688.50 | 15.60% | Pons et al., 2013 |
| Potato | Vitamin A | β-carotene | 0.0491 | 1 tuber (173 g) | 4,567 | 42.30% | Diretto et al., 2007 |
| Rice | Vitamin A | β-carotene | 0.0689 | 100 g edible portion | 3,700 | 34.30% | Ye et al., 2000 |
| Sorghum | Vitamin A | β-carotene | 0.0745 | 100 g edible portion | 4,000 | 37.00% | Lipkie et al., 2013 |
| Tomato | Vitamin A | β-carotene | 0.0838 | 1 medium fruit (~123 g) | 5,535 | 51.20% | Li et al., 2018 |
| Tomato | Vitamin A | β-carotene | 1.1179 | 1 medium fruit (~123 g) | 73,800 | 683.30% | Liu et al., 2025 |
| Rice | Vitamin B1 | thiamin | 9.07 × 10⁻⁴ | 100 g edible portion | 306 | 25.50% | Strobbe et al., 2021 |
| Potato | Vitamin B6 | pyridoxine equivalent | 0.0355 | 1 medium tuber (~173 g) | 1,038 | 79.80% | Bagri et al., 2018 |
| Rice | Vitamin B6 | pyridoxine equivalent | 0.0649 | 100 g edible portion | 1,100 | 84.60% | Mangel et al., 2019 |
| Wheat | Vitamin B6 | pyridoxine equivalent | 4.14 × 10⁻⁴ | 100 g edible portion | 70 | 5.40% | Mohsin et al., 2022 |
| Lettuce | Vitamin B9 | folate | 0.793 | 50 g edible portion | 17,500 | 4375.00% | Nunes et al., 2009 |
| Maize | Vitamin B9 | folate | 0.0044 | 100 g edible portion | 194 | 48.50% | Naqvi et al., 2009 |
| Rice | Vitamin B9 | folate | 0.0074 | 100 g edible portion | 325 | 81.20% | Blancquaert et al., 2015 |
| Rice | Vitamin B9 | folate | 0.0042 | 100 g edible portion | 185 | 46.20% | Storozhenko et al., 2007 |
| Tomato | Vitamin B9 | folate | 0.0041 | 1 medium fruit (~123 g) | 217.2 | 54.30% | Díaz de la Garza et al., 2004 |
| Maize | Vitamin C | ascorbic acid | 0.607 | 100 g edible portion | 10,694 | 11.90% | Naqvi et al., 2009 |
| Potato | Vitamin C | ascorbic acid | 9.369 | 1 medium tuber (~173 g) | 285,450 | 317.20% | Bulley et al., 2012 |
| Strawberry | Vitamin C | ascorbic acid | 6.302 | 1 cup halves (~144 g) | 159,840 | 177.60% | Bulley et al., 2012 |
| Tomato | Vitamin C | ascorbic acid | 10.391 | 1 medium fruit (~123 g) | 225,090 | 250.10% | Bulley et al., 2012 |
| Rice | Zinc | Zn | 0.535 | 100 g edible portion | 3,500 | 31.80% | Lee et al., 2009 |
| Rice | Zinc | Zn | 0.535 | 100 g edible portion | 3,500 | 31.80% | Masuda et al., 2007 |
| Rice | Zinc | Zn | 0.535 | 100 g edible portion | 3,500 | 31.80% | Wirth et al., 2009 |
| Rice | Zinc | Zn | 1.163 | 100 g edible portion | 7,600 | 69.10% | Johnson et al., 2011 |
| Values represent nutrient concentrations expressed in µmol g⁻¹ and converted to estimated intake (µg) based on typical consumption portions for each crop. The percentage of adult RDA met was calculated using standard dietary reference values and reflects the contribution of a single serving of the biofortified crop to daily nutrient requirements. Analytes correspond to the measured nutrient forms (e.g., Fe, β-carotene, thiamin, pyridoxine equivalents, folate). Usual consumption values were defined based on commonly reported edible portions (e.g., 100 g grains, one medium fruit, or one tuber). Data were compiled from published studies as indicated. | | | | | | | |

| **Table S2. Dietary anthocyanin intake thresholds associated with vascular, metabolic, and anti-inflammatory health outcomes** | | |
| --- | --- | --- |
| **Threshold** | **What it represents** | **Evidence / typical finding** |
| **20 mg/day** | Minimum intake associated with **improved vascular function** | Better endothelial function and blood flow |
| **22 mg/day** | Intake associated with **reduced risk of type 2 diabetes** in large cohorts | Observational dietary studies |
| **200 mg/day** | Intake linked to **significant cardiometabolic improvements** | Reduced LDL cholesterol, improved HDL, reduced oxidative stress |
| **> 300 mg/day** | Intake associated with **reduction in inflammatory biomarkers** | Lower CRP, IL-6, TNF-α, ICAM-1, VCAM-1 |

Key Studies Establishing These Thresholds

1. **Diabetes risk threshold (~22 mg/day)**

Guo et al., 2016 – American Journal of Clinical Nutrition

Prospective cohort studies

Anthocyanin intake ≈ 22 mg/day

Associated with lower type 2 diabetes incidence

**2. Vascular function threshold (20–40 mg/day)**

Wallace et al., 2016 – Advances in Nutrition

Human intervention trials

20–40 mg/day anthocyanins

Improved endothelial function and vascular reactivity

**3. Cardiometabolic benefits (200–400 mg/day)**

Sandoval-Ramírez et al., 2022 – Antioxidants

Meta-analysis of clinical trials

200–400 mg/day

Improvements in:

LDL cholesterol

HDL cholesterol

insulin sensitivity

**4. Anti-inflammatory effects (> 300 mg/day)**

Fallah et al., 2020 – Clinical Nutrition

Randomized controlled trials

> 300 mg/day

Reduced:

CRP

IL-6

TNF-α

ICAM-1

VCAM-1

Thresholds represent approximate daily intake levels linked to improvements in vascular function, reduced risk of type 2 diabetes, cardiometabolic health, and inflammation. Reported outcomes include changes in endothelial function, lipid profiles, insulin sensitivity, and circulating inflammatory biomarkers such as C-reactive protein (CRP), interleukin-6 (IL-6), tumor necrosis factor-α (TNF-α), intercellular adhesion molecule-1 (ICAM-1), and vascular cell adhesion molecule-1 (VCAM-1). Supporting evidence is derived from key studies including prospective cohort analyses (e.g., Guo et al., 2016), controlled human trials (e.g., Wallace et al., 2016), and meta-analyses of randomized clinical trials (e.g., Sandoval-Ramírez et al., 2022; Fallah et al., 2020).

| **Table S3. Estimated anthocyanin intake from biofortified crops relative to reported dietary thresholds for health benefits.** | | | | | | | | | |
| --- | --- | --- | --- | --- | --- | --- | --- | --- | --- |
| **Crop** | **Gene** | **Anthocyanin content** | **Typical consumption** | **Intake per serving (mg)** | **% of 20 mg threshold** | **% of 22 mg threshold** | **% of 200 mg threshold** | **% of 300 mg threshold** | **Reference** |
| Grapevine | VvbZIP36 | 0.359 mg/g | 100 g leaves | **35.9 mg** | 180% | 163% | 18% | 12% | Tu et al., 2022 |
| Rice | ZmLc; ZmPl; SsCHS; SsCHI; SsF3H; SsF3′H; SsDFR; SsANS | 1 mg/g | 100 g grain | **100 mg** | 500% | 455% | 50% | 33% | Zhu et al., 2017 |
| Strawberry | FaMYB5 | 8 µg/g | 100 g fruit | **0.8 mg** | 4% | 3.60% | 0.40% | 0.27% | Zhang et al., 2023 |
| Tomato | Delila; Rosea1 | 2.83 mg/g DW (~0.283 mg/g FW) | 100 g fruit | **28.3 mg** | 142% | 129% | 14% | 9% | Butelli et al., 2008 |
| Wheat | ThMYC4E | 0.5 mg/g | 100 g grain | **50 mg** | 250% | 227% | 25% | 17% | Zhao et al., 2019 |
